# Comparative proteomics reveals that central metabolism changes are associated with resistance against *Sporisorium scitamineum* in sugarcane

**DOI:** 10.1186/s12864-016-3146-8

**Published:** 2016-10-12

**Authors:** Yachun Su, Liping Xu, Zhuqing Wang, Qiong Peng, Yuting Yang, Yun Chen, Youxiong Que

**Affiliations:** 1 Key Laboratory of Sugarcane Biology and Genetic Breeding, Ministry of Agriculture, Fujian Agriculture and Forestry University, Fuzhou, 350002 China; 2 Guangxi Collaborative Innovation Center of Sugarcane Industry, Guangxi University, Nanning, 530005 China

**Keywords:** *Saccharum* spp., *Sporisorium scitamineum*, iTRAQ, RNA-seq, Transcriptome, Proteome

## Abstract

**Background:**

Sugarcane smut, which is caused by *Sporisorium scitamineum*, has been threatening global sugarcane production. Breeding smut resistant sugarcane varieties has been proven to be the most effective method of controlling this particular disease. However, a lack of genome information of sugarcane has hindered the development of genome-assisted resistance breeding programs. Furthermore, the molecular basis of sugarcane response to *S. scitamineum* infection at the proteome level was incomplete and combining proteomic and transcriptional analysis has not yet been conducted.

**Results:**

We identified 273 and 341 differentially expressed proteins in sugarcane smut-resistant (Yacheng05-179) and susceptible (ROC22) genotypes at 48 h after inoculation with *S. scitamineum* by employing an isobaric tag for relative and absolute quantification (iTRAQ). The proteome quantitative data were then validated by multiple reaction monitoring (MRM). The integrative analysis showed that the correlations between the quantitative proteins and the corresponding genes that was obtained in our previous transcriptome study were poor, which were 0.1502 and 0.2466 in Yacheng05-179 and ROC22, respectively, thereby revealing a post-transcriptional event during Yacheng05-179-*S. scitamineum* incompatible interaction and ROC22-*S. scitamineum* compatible interaction. Most differentially expressed proteins were closely related to sugarcane smut resistance such as beta-1,3-glucanase, peroxidase, pathogenesis-related protein 1 (PR1), endo-1,4-beta-xylanase, heat shock protein, and lectin. Ethylene and gibberellic acid pathways, phenylpropanoid metabolism and PRs, such as PR1, PR2, PR5 and PR14, were more active in Yacheng05-179, which suggested of their possible roles in sugarcane smut resistance. However, calcium signaling, reactive oxygen species, nitric oxide, and abscisic acid pathways in Yacheng05-179 were repressed by *S. scitamineum* and might not be crucial for defense against this particular pathogen.

**Conclusions:**

These results indicated complex resistance-related events in sugarcane-*S. scitamineum* interaction, and provided novel insights into the molecular mechanism underlying the response of sugarcane to *S. scitamineum* infection.

**Electronic supplementary material:**

The online version of this article (doi:10.1186/s12864-016-3146-8) contains supplementary material, which is available to authorized users.

## Background

Smut of sugarcane (*Saccharum* spp.), which is caused by the fungus *Sporisorium scitamineum*, has been reported worldwide, mainly in sugarcane planting areas [1]. Infected plants present an elongated whip, profuse tillering, thin stalks, and small narrow leaves. Hence, this infection can result in substantial yield loss and quality reduction [1, 2]. Breeding smut resistant sugarcane varieties has been proven to be the most effective method of controlling this particular disease [1, 2]. However, due to its complex polyploid-aneuploid genome background (more than 120 chromosomes) and the complicated interaction between smut pathogen, and the environment, at least 10 years of multi-point resistance and productivity evaluation testing is required in order to release smut resistant sugarcane variety [3, 4]. To date, whole genome sequencing of sugarcane has not been completed by virtue of its huge genome size (about 10 Gb), which in turn limits the development of genome-assisted breeding programs [4]. In 2014, the *S. scitamineum* genome (19.8 Mb) was obtained by our laboratory and has provided insights on the pathogenic mechanisms of the sugarcane smut fungus [5].

Previous researches have mainly focused on the cytology [1], morphology [6], physiology, biochemistry [7], as well as genetics of sugarcane [8], to explore smut resistant mechanisms. Recent reports have described the molecular basis of sugarcane response to *S. scitamineum* infection, and several differentially expressed genes were identified by Thokoane and Rutherford [9], Borrás-Hidalgo et al. [10], and Que et al. [11] using a cDNA-amplified fragment length polymorphism (cDNA-AFLP) method. Two of our previous studies were focused on the changes in gene expression profiles in sugarcane challenged by *S. scitamineum* using high-throughput sequencing [12, 13]. Wu et al. [12] used a high-throughput tag-sequencing Solexa sequencing technology to analyze the transcriptome profile in sugarcane post-*S. scitamineum* inoculation, and 2015 differentially expressed genes were identified, including 1125 upregulated and 890 downregulated genes. Que et al. [13] performed transcriptome analysis by RNA sequencing (RNA-seq) of smut-resistant and susceptible sugarcane genotypes (Yacheng05-179 and ROC22) after *S. scitamineum* infection. Bioinformatics analysis revealed 65,852 unigenes, of which more transcripts associated with resistance in Yacheng05-179 (24–48 h) were induced earlier than that in ROC22 (48–120 h), thereby revealing resistance specificity and early timing of resistance genes in the incompatible interaction. These studies have demonstrated that the molecular mechanism of sugarcane response to smut pathogen infection is complex [12, 13].

Due to post-transcriptional regulation and translational processes, transcripts are not always consistent well with their final products (proteins) [14, 15]. Protein analysis, which describes more direct molecular responses than conventional genomics, is necessary to better enhance our understanding of plant immunity. Isobaric tags for relative and absolute quantitation (iTRAQ), which involves a single sensitive mass spectrometry (MS) analysis with multiple samples, has been successfully adopted in quantitative proteomics [16, 17]. This technique can more accurately assess and quantify protein levels, as well as reduce experimental errors generated by individual experiments [16]. Recently, several advances in identifying proteins associated with the pathogenic process have been performed by using the iTRAQ method [17, 18]. Wang et al. [17] identified 260 and 183 specifically accumulated proteins in Yuyan8 and NC89 at 24 h during *Nicotiana tabacum*-tobacco mosaic virus (TMV) interaction using the iTRAQ method, respectively. Parker et al. [18] determined by using the iTRAQ approach that 477 of 2369 expressed proteins in tomato were responsive to *Pseudomonas syringae* inoculation. Multiple reaction monitoring (MRM) with high-throughput confirmation through measurements of representative peptides via MS has become a powerful method for quantifying targeted proteomics [19, 20]. Currently, iTRAQ assay combined with subsequent MRM confirmation has been adopted to determine key protein biomarkers in diseases [21, 22]. From 1970 to 2014, one-dimensional gradient polyacrylamide gels (1DE), 2DE and MS methods have been utilized to analyze the sugarcane proteome under various abiotic and biotic stresses [23] such as drought [24, 25], salt [26, 27], osmosis [28, 29], *Gluconacetobacter diazotrophicus* [30], *Xanthomonas albilineans* [31], and *S. scitamineum* [32] stimuli. Que et al. [32] detected 20 differentially expressed proteins that were related to signal transduction, photosynthesis, or disease resistance in sugarcane post *S. scitamineum* inoculation by using 2DE and matrix-assisted laser desorption/ionization time of flight mass spectrometry (MALDI-TOF-TOF/MS) methods. Although this study has laid a foundation for understanding the response of sugarcane to *S. scitamineum* infection at the proteome level, the information was incomplete and combining proteomic and transcriptional analysis has not yet been conducted.

The smut pathogen enters the meristem of the buds between 6 and 36 h after teliospore deposition [33]. Que et al. [13] reported that the number of differentially expressed genes in Yacheng05-179 (smut-resistant) at 48 h after *S. scitamineum* inoculation was higher than that at 24 and 120 h, which also exceeded the number of differentially expressed genes in ROC22 (smut-susceptible) at the same time point (48 h). In the present study, iTRAQ technology combined with MRM assay, was employed to characterize the proteome changes in both smut-resistant sugarcane genotype Yacheng05-179 and smut-susceptible sugarcane genotype ROC22 post-*S. scitamineum* infection at 48 h. Furthermore, because these were derived from exactly the same experiment, an integrated analysis of its proteome and transcriptome was also conducted. The transcriptome data were obtained from our previous work [13]. The aim of the present study was to reveal the central metabolism changes in sugarcane against *S. scitamineum*.

## Methods

### Plant materials and inoculation with *S. scitamineum*

The sugarcane genotypes of Yacheng05-179 (smut resistant) and ROC22 (smut susceptible), as well as smut whips from the most popular cultivar ROC22, were collected from the Key Laboratory of Sugarcane Biology and Genetic Breeding, Ministry of Agriculture (Fuzhou, China). Two-bud setts of both sugarcane genotypes were grown at 28 °C with condition of 12 h light and 12 h dark photoperiod, after inoculation with 0.5 μL suspension containing 5 × 10^6^/mL *S. scitamineum* spores (with 0.01 % volume ratio of Tween-20), whereas control buds were syringe-inoculated with sterile distilled water (with 0.01 % volume ratio of Tween-20) [13]. The buds from both genotypes were collected at 0, 24, 48 and 120 h after inoculation, immediately frozen in liquid nitrogen, and then stored at −80 °C until further analysis. Half of the samples were used to extract the protein, and another aliquot was used for RNA extraction.

### Protein extraction, digestion, and iTRAQ labeling

Because the present plant materials was derived from exactly the same experiment as that of our previous transcriptome report [13], the harvested buds from Yacheng05-179 and ROC22 inoculated with distilled water (named YCK and RCK, respectively) and *S. scitamineum* at 48 h (named YT and RT, respectively), were used for protein extraction according to the protocol that integrated trichloroacetic acid (TCA)/acetone precipitation with a methanol wash and phenol extraction, respectively [34]. The protein concentration was determined by using the Bradford’s method using bovine serum albumin (BSA) as standard [34]. Total protein (100 μg) from each sample solution, was trypsin-digested following Wu et al. [35]. iTRAQ analysis was conducted at the Beijing Genomics Institute (BGI, Shenzhen, China). Five biological replicates were pooled for iTRAQ analysis. Samples of YCK, YT, RCK and RT were labeled with iTRAQ reagents with molecular masses of 113, 115, 117 and 119 Da by iTRAQ Reagent-8plex Multiplex Kit (Applied Biosystems, Foster City, CA, USA), respectively.

### Strong cation exchange (SCX) fractionation

For SCX chromatography utilizing a LC-20AB high performance liquid chromatography (HPLC) pump system (Shimadzu, Japan), the iTRAQ-labeled peptides were reconstituted with 4 mL of buffer A (25 mM NaH_2_PO_4_ in 25 % acetonitrile, pH 2.7) and loaded onto a 4.6 × 250 mm Ultremex SCX column. The peptides were eluted across a gradient at a flow rate of 1 mL/min as follows: 5 % buffer B (25 mM NaH_2_PO_4_, 1 M KCl in 25 % acetonitrile, pH 2.7) for 7 min, 60 % buffer B for 20 min, 100 % buffer B for 1 min, then maintained in 5 % buffer B for 10 min. Elution was monitored by measuring the absorbance at a wavelength of 214 nm, and the eluted peptides were pooled into 12 fractions. After that, they were then desalted by using a column of Luna 5u SCX 100A 250 × 4.6 mm (Phenomenex, USA) and then dried by vacuum centrifugation [35].

### Liquid chromatography-electrospray in trap tandem mass spectrometry (LC-ESI-MS/MS) analysis by TripleTOF 5600

Each of the dried fractions was resuspended in buffer A (5 % acetonitrile, 0.1 % formic acid) and then centrifuged at 20,000 g for 10 min. A five-microliter fraction (approximately 2.5 μg of protein) was loaded into a 2 cm C18 trap column (inner diameter: 200 μm) on a Shimadzu LC-20 AD nano HPLC. The samples were loaded at 8 μL/min for 4 min, then run at 300 nL/min in 5 % buffer B (95 % acetonitrile, 0.1 % formic acid) for 5 min, followed by the gradient treatment run from 5 to 35 % buffer B for 35 min, and by a 5 min linear gradient to 60 %, maintenance at 80 % buffer B for 2 min, and finally a return to 5 % buffer B for 10 min. The eluted peptides were subjected to nanoelectrospray ionization followed by MS/MS in a mass spectrometer of TripleTOF 5600 (AB SCIEX, Concord, ON, Canada) fitted with a Nanospray III source (AB SCIEX) and a pulled quartz tip as the emitter (New Objectives, Woburn, MA, USA) [35].

Data was acquired using an ion spray voltage of 2.5 kV, curtain gas of 30 psi, nebulizer gas of 15 psi, and an interface heater temperature of 150 °C. The MS was operated with a RP of ≥ 30,000 FWHM for TOF MS scans. For information-dependent acquisition (IDA), survey scans were acquired in 250 ms and as many as 30 product ion scans were collected when exceeding a threshold of 120 counts per second and with a 2+ to 5+ charge states. Total cycle time was fixed to 3.3 s. Q2 transmission window was 100 Da for 100 %. Four time bins were summed for each scan at a pulser frequency value of 11 kHz through monitoring of the 40 GHz multichannel TDC detector with four-anode channel detection. A sweeping collision energy setting of 35 ± 5 eV coupled with iTRAQ adjust rolling collision energy was applied to all precursor ions for collision-induced dissociation. Dynamic exclusion was set for 1/2 of peak width (15 s), and then the precursor was refreshed off the exclusion list.

### Protein identification and quantification

The software Mascot 2.3.02 (Matrix Science, UK) was employed for protein analysis. Sugarcane_Unigene (65,852 unigenes) derived from our previous transcriptome analysis at 24 h, 48 h, and 120 h post-*S. scitamineum* infection was used as search database [13]. Spectra from the 12 fractions were combined into one Mascot generic format (MGF) file after the raw data were loaded. Then the MGF file was searched by the parameters as follows: trypsin as enzyme; Gln- > Pyro-Glu (N-term Q), Oxidation (M), iTRAQ8plex (Y) as the variable modifications; Carbamidomethyl (C), iTRAQ8plex (N-term), iTRAQ8plex (K) as fixed modifications; the fragment and peptide mass tolerance were set as 0.1 Da and 0.05 Da, respectively. An automatic decoy database search strategy was used to estimate the false discovery rate (FDR). The FDR was calculated as the false positive matches divided by the total matches. In the final search results, the FDR was less than 1 %. For protein identification, the filters were set as follows: significance threshold *P*, 0.05 (with 95 % confidence) and ion score or expected cutoff less than 0.05 (with 95 % confidence). For protein quantitation, iTRAQ labeled peptides was quantified with Mascot 2.3.02 using the isotopic corrections, and the parameters were set as follows: (i) protein ratio type was set as “weighted”; (ii) median intensities were chosen for normalization; (iii) minimum peptides were set to two; (iv) only unique peptides were selected to quantify proteins. Ratios of the same protein among different spectras were automatically executed based on the two-tailed *t*-test method by the Mascot 2.3.02 software. A ratio with *P*-value < 0.05, fold change > 1.20 (upregulated) or < 0.83 (downregulated) [18, 36–38] were considered as significantly differentially expressed proteins. In this study, three comparisons of YT vs. YCK (YT/YCK), RT vs. RCK (RT/RCK), and YT/YCK vs. RT/RCK were performed. For Gene Ontology (GO) classification analysis (http://www.geneontology.org) and Kyoto Encyclopedia of Genes and Genomes (KEGG) enrichment analysis (http://www.kegg.jp/), the homology search was performed for all query protein matches with BLASTP against the Sugarcane_Unigene database. In addition, combined with the data of proteome and our previous transcriptome (fold change ≥ 2 and FDR ≤ 0.01) [13] under the same treatment condition, correlation analysis was performed. When a certain amount of protein was expressed at the transcript level based on the identification results of the proteome and transcriptome, this served as a correlated differentially expressed protein. The correlation between the proteins and transcripts, which were identified, quantified or differentially expressed, was calculated by using Pearson’s correlation coefficient. In addition, the numbers of correlated differential proteins that have similar or inverse expression trends at the transcript level were also counted.

### Protein validation by MRM

MRM performance was evaluated for five differentially expressed proteins from iTRAQ. Details of the transitions selection and MRM method validation were described in Additional file 1: Text S1. The Skyline software [39] was used to select peptides of the target proteins with a MS/MS spectral library (cut-off score > 0.95) which was generated on a TripleTOF5600 (AB SCIEX, Foster City, CA) and searched using Mascot v2.3 (Matrix Science, UK) against with a *Saccharum* database (72,441 entries). The sugarcane buds from Yacheng05-179 and ROC22 inoculated with distilled water (named YCK and RCK) and *S. scitamineum* at 48 h (named YT and RT) were regarded as control and treatment samples, respectively. Three biological replicates for both the treatment (YT1, YT2, and YT3; RT1, RT2, and RT3) and control (YCK1, YCK2, and YCK3; RCK1, RCK2, and RCK3) were used for protein extraction according to the protocol that integrated TCA/acetone precipitation with a methanol wash and phenol extraction [34]. Total protein (100 μg) was taken out of each sample solution and digested with Trypsin Gold (Promega, Madison, WI, USA) with the ratio of protein:trypsin =30:1 at 37 °C for 16 h. Then the peptides were dried by vacuum centrifugation and reconstituted in 0.5 M tetraethyl-ammonium bromide (TEAB, Applied Biosystems, Milan, Italy). Samples were spiked with 50 fmol of beta-galactosidase (P00722) for data normalization. MRM analysis was performed on a QTRAP 5500 mass spectrometer (AB SCIEX, Foster City, CA) equipped with a LC-20 AD nano HPLC system (Shimadzu, Kyoto, Japan). The mobile phase consisted of 0.1 % aqueous formic acid (solvent A) and 98 % acetonitrile with 0.1 % formic acid (solvent B). Peptides were separated on a BEH130 C18 column (0.075 × 150 mm column, 3.6 μm; Waters) at 300 nL/min, and eluted with a gradient of 5 % − 30 % solvent B for 38 min, 30 % − 80 % solvent B for 4 min, and maintenance at 80 % for 8 min. For the QTRAP 5500 mass spectrometer, spray voltage of 2400 V, nebulizer gas of 23 p.s.i., and a dwell time of 10 ms were used. Multiple MRM transitions were monitored using a unit resolution in both Q1 (the mass to charge ratio of the parent ion) and Q3 (the mass to charge ratio of the product ion) quadrupoles to maximize specificity. Skyline software was applied to integrate the raw file generated by QTRAP 5500. The iRT strategy was used to define a chromotography of a given peptide against the spectral library [40]. All transitions for each peptide was used for quantitation unless interference from the matrix was observed. The beta-galactosidase peptides were used as internal standards for relative quantification of protein levels. MSstats with the linear mixed-effects model were used [41]. The *P*-value was adjusted to control the FDR at a cutoff of 0.05. All proteins with a *P*-value < 0.05 and a fold change > 1.2 were considered significant. All MRM analyses were run in triplicate.

### Reverse transcription quantitative real-time polymerase chain reaction (RT-qPCR) analysis

To investigate the expression patterns of the associated genes/proteins between transcriptome and proteome data, as well as a series of induced proteins in the calcium, reactive oxygen species (ROS), nitric oxide (NO), abscisic acid (ABA), ethylene (ET), and gibberellic acid (GA) pathways, the time points of 0 h, 24 h, 48 h and 120 h during Yacheng05-179-*S. scitamineum* incompatible interaction and ROC22-*S. scitamineum* compatible interaction were selected as samples in the RT-qPCR analysis. A total of 22 genes (Additional file 2: Table S1) were selected in designing gene-specific primers for RT-qPCR validation. The glyceraldehyde-3-phosphate dehydrogenase (*GAPDH*) gene served as the internal reference gene [42]. SYBR Green was applied for RT-qPCR in the ABI 7500 fast real-time PCR system (Applied Biosystems, Foster, CA, USA). RT-qPCR was conducted out in a 20 μL reaction mixture containing 10 μL FastStart Universal SYBR Green PCR Master (ROX), 0.5 μmol of each primer and 1.0 μL template (20 × diluted cDNA). RT-qPCR conditions were as follows: 50 °C, 2 min; 95 °C, 10 min; followed by 40 cycles of 95 °C, 15 s and 60 °C, 1 min. Three replicates were performed for each sample. A PCR using distilled water as template was used as negative control. The 2^-△△Ct^ method was adopted for quantitative gene expression analysis [43]. Statistical analysis was conducted using the Data Processing System (DPS) v7.05 software (China). Data were expressed as the mean ± standard error (SE). Significance (*P*-value < 0.05) was calculated using the one-way Analysis of Variance (ANOVA) followed by multiple Duncan tests.

### Role of the correlated protein of beta-1,3-glucanase in response to pathogen infection

Beta-1,3-glucanase (Sugarcane_Unigene_BMK.34407, abbreviated as SU34407), a pathogenesis-related protein (PR), was identified in Yacheng05-179 at both the transcript and protein levels but remained unchanged in ROC22. Amino acid sequence alignment showed that the sequence of SU34407 was consistent with that of ScGluA1 (GenBank Accession No. KC848050) that was described in our previous study [44]. The overexpression vector pCAMBIA 1301-*ScGluA1* was constructed and transformed into *Agrobacterium* strain EHA105. Then, the cultured cells, *Agrobacterium* strain EHA105 containing pCAMBIA 1301 vector alone (*35S::00*) or pCAMBIA 1301-*ScGluA1* (*35S::ScGluA1*) were diluted in MS liquid medium (containing 200 μM acetosyringone) to OD_600_ = 0.8 and infiltrated into the eight-leaf stage-old *N. benthamiana* leaves. All plants were cultivated with 28 °C in condition of 16 h light and 8 h dark for 1 d. Then the cultured cells (OD_600_ = 0.5) of *N. tabacum Fusarium solani* var. *coeruleum* or *Botrytis cinerea*, which were diluted in 10 mM magnesium chloride (MgCl_2_), were respectively infiltrated into the main vein of the infected leaves. These plant materials were cultivated using the same conditions for 20 d and photographed [13].

Transgenic *N. benthamiana* plants were infected with *Agrobacterium* strain EHA105 carrying the pCAMBIA 1301 vector alone (*35S::00*) or pCAMBIA 1301-*ScGluA1* (*35S::ScGluA1*) through the leaf disc method and identified by PCR and RT-PCR (Additional file 3: Figure S1), respectively. The antimicrobial action of the beta-1,3-glucanase enzyme from transgenic *ScGluA1 N. benthamiana* leaves on the hyphal growth of *F. solani* var. *coeruleum* were validated by using a filter paper assay. The mycelia of the *F. solani* var. *coeruleum* were inoculated in the middle of the potato dextrose agar (PDA) medium and cultivated at 28 °C for 4 d. Then the filter papers at around 1 cm distance from hyphae were filled with beta-1,3-glucanase enzyme from three different T_0_ generation transgenic *35S::ScGluA1 N. benthamiana* plants, whereas the control was filled with beta-1,3-glucanase enzyme from T_0_ generation of transgenic *35S::00* or non-transgenic *N. benthamiana* plants, or 0.05 M sodium acetate buffer (pH 5.0), respectively. The antimicrobial effects were evaluated by visual inspection after cultivation at 28 °C for 2 d and 4 d [13]. The functional analysis of *ScGluA1* here shared the same controls as that of *ScChi* in our previous report [13], which were derived from exactly the same experiment.

## Results

### iTRAQ protein profiling

A total of 17,634 unique peptides and 4251 proteins (at least one unique peptides with high confidence) (Additional file 4: Table S2) were identified by iTRAQ analysis against the Sugarcane_Unigene database. Among these, 3696 proteins were annotated to 46 GO terms by GO analysis. In terms of biological process categories, most proteins were categorized into the metabolic process (19.27 %), cellular process (17.91 %) and single-organism process (10.26 %). The GO term percentages of response to stimulus and immune system process were 8.87 and 0.87 %, respectively. The major cellular components were cell (24.21 %) and cell part (24.21 %). The largest molecular functions of proteins obtained by GO analysis were catalytic activity (44.87 %) and binding (41.93 %). Using pathway analysis, 2884 proteins were annotated to 127 pathways. Table 1 showed the top 10 pathways with the largest number of proteins.

**Table 1:** The top ten pathways with the largest number of proteins

No	Pathway	Count (2884)	Pathway ID
1	Metabolic pathways	886	ko01100
2	Biosynthesis of secondary metabolites	529	ko01110
3	Ribosome	128	ko03010
4	Spliceosome	113	ko03040
5	Protein processing in endoplasmic reticulum	100	ko04141
6	Phenylpropanoid biosynthesis	97	ko00940
7	Plant-pathogen interaction	94	ko04626
8	RNA transport	93	ko03013
9	Plant hormone signal transduction	87	ko04075
10	Purine metabolism	84	ko00230

### Identification of differentially abundant proteins post *S. scitamineum* infection

**Fig. 1:**
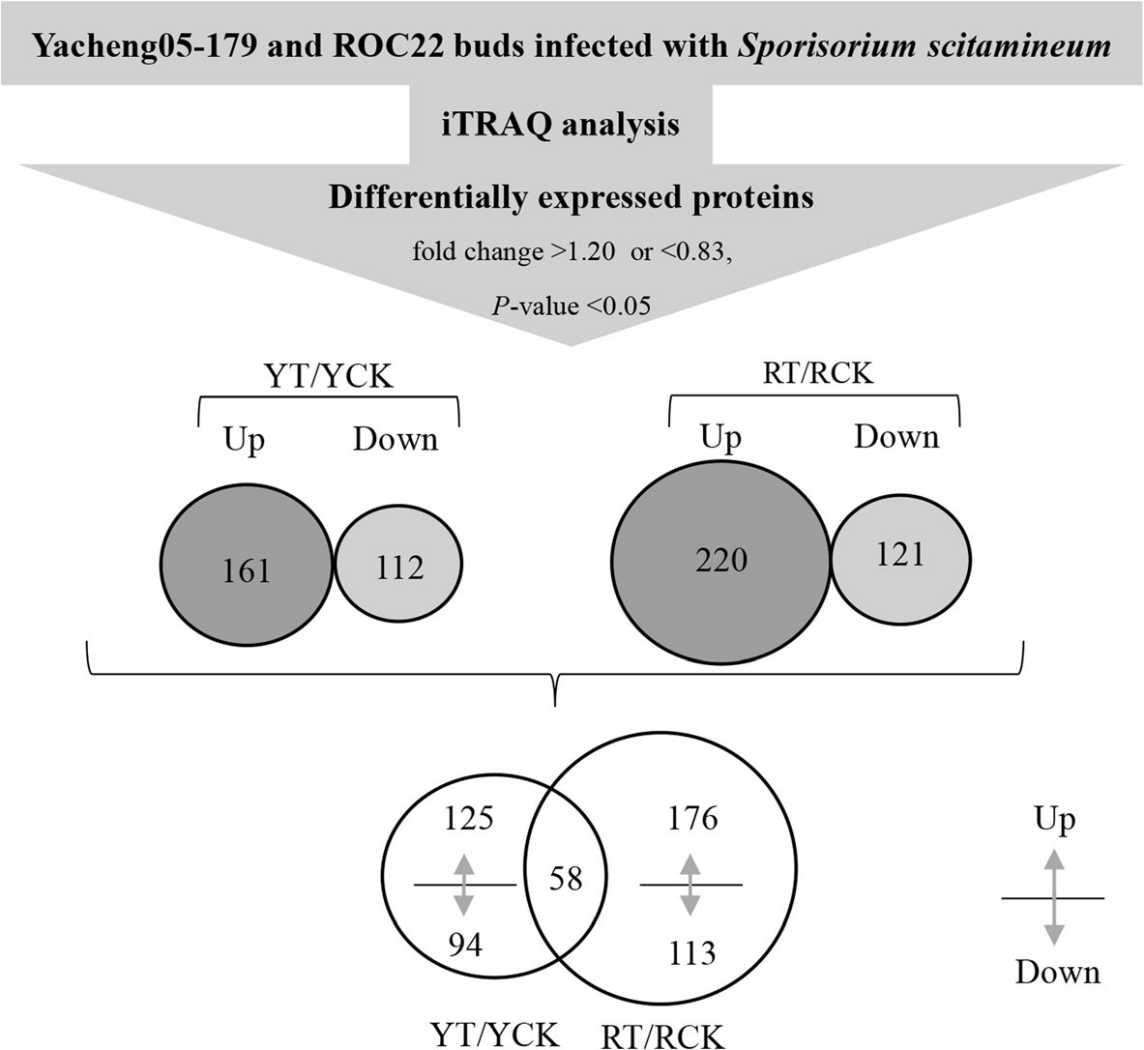
Distribution of differentially expressed proteins between resistant and susceptible genotypes. Ratios with *P*-value < 0.05, fold change > 1.20 (upregulated) or < 0.83 (downregulated) are considered significantly differentially expressed. YCK and YT: Yacheng05-179 under sterile water and *Sporisorium scitamineum* stresses after 48 h, respectively; RCK and RT: ROC22 under sterile water and *S. scitamineum* stresses after 48 h, respectively

In the present study, proteome changes between the two sugarcane genotypes in response to pathogen challenge were investigated (Fig. 1). Compared to the control group, a 1.20-fold or 0.83-fold change threshold with a *P*-value < 0.05 in protein expression were classified as a physiologically significant change, and 273 proteins were quantified to be differentially expressed in Yacheng05-179-*S. scitamineum* interaction by iTRAQ analysis, including 161 upregulated (Additional file 5: Table S3) and 112 downregulated proteins (Additional file 6: Table S4). A total of 341 proteins were differentially expressed in ROC22-*S. scitamineum* interaction, of which 220 were upregulated (Additional file 7: Table S5) and 121 were downregulated (Additional file 8: Table S6). In the Venn diagram, 58 differentially expressed proteins were shared between these two genotypes. The shared differentially expressed proteins in Yacheng05-179 contained 40 upregulated and 18 downregulated proteins, whereas those in ROC22 included 50 upregulated and 8 downregulated proteins. The unique differentially expressed proteins in Yacheng05-179 contained 125 upregulated and 94 downregulated proteins, whereas those in ROC22 included 176 upregulated and 113 downregulated proteins.

### Correlation of protein fold changes with transcripts

**Fig. 2:**
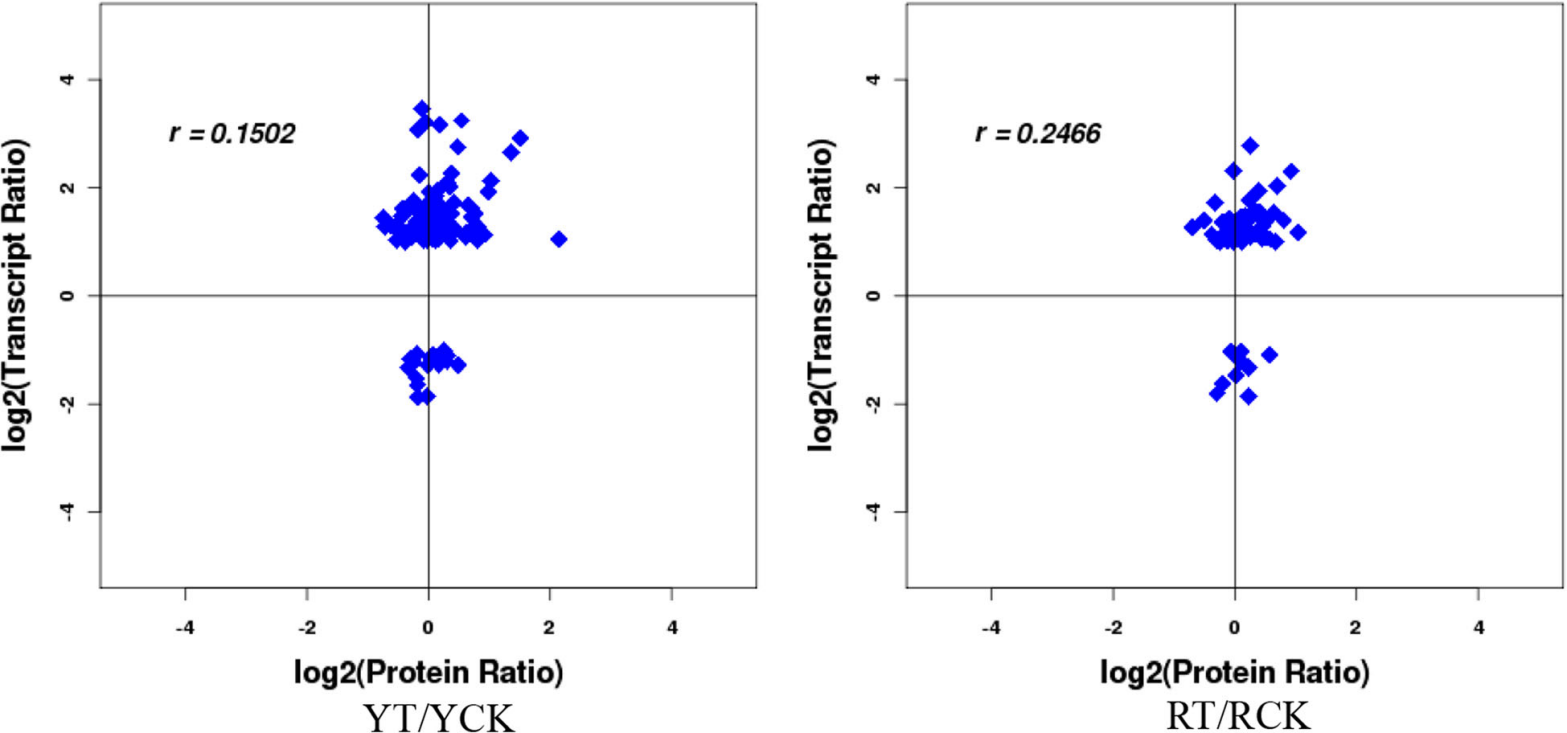
Association graphs of the quantitative proteins with their corresponding genes. YCK and YT: Yacheng05-179 under sterile water and *Sporisorium scitamineum* stresses after 48 h, respectively; RCK and RT: ROC22 under sterile water and *S. scitamineum* stresses after 48 h, respectively. The bottom circles in the four quadrants represent the expression trends of the differentially expressed proteins that are associated with the transcriptome. a quadrant, correlated proteins/genes upregulated in both proteome and transcriptome. b quadrant, correlated proteins/genes upregulated in the proteome and downregulated in the transcriptome. c quadrant, correlated proteins/genes downregulated in both proteome and transcriptome. d quadrant, correlated proteins/genes downregulated in the proteome and upregulated in the transcriptome

In a parallel analysis, RNA-seq information, which was obtained from exactly the same experiment in our previous report [13], was compared to the proteins data. In the present study, 4038 of 25,735 and 4034 of 25,542 identified transcripts for which corresponding proteins were represented in the iTRAQ-based proteomics were determined in YT/YCK and RT/RCK, respectively. Furthermore, the number of correlated quantified mRNAs and proteins were 115 and 60 in YT/YCK and RT/RCK, respectively. The distribution of the corresponding mRNA:protein ratios was presented using a scatter plot (Fig. 2). Figure 2 showed that most of the quantified mRNA and their corresponding protein levels did not vary above 2-fold. The correlation between the quantified differential transcripts and proteins was 0.1502 and 0.2466 in YT/YCK and RT/RCK, respectively. Of the 273 quantified differential proteins, 27 were associated with transcriptome in YT/YCK, whereas 10 out of 341 differential proteins had corresponding transcripts in the RNA-seq data in RT/RCK (Table 2). Among these, 18 and 7 correlated proteins, which have the similar gene expression trend at the transcript level, increased in YT/YCK and RT/RCK, respectively. Interestingly, most correlated differential proteins were closely related to plant stress resistance such as SU34407, peroxidase (POD) (SU59640, gi34949353, SU58110 and SU50541), PR1 (SU51436, and gi36048114), endo-1,4-beta-xylanase (SU55512), heat shock protein (HSP) (SU42413 and SU42924), and lectin (SU84564).

**Table 2:** Analysis of differentially expressed proteins associated with transcriptome

Protein ID	Annotation	YT/YCK			RT/RCK		
		Proteins (fold change)	Transcripts (log_2_ fold change)	FDR	Proteins (fold change)	Transcripts (log_2_ fold change)	FDR
Sugarcane_Unigene_BMK.34407	Beta-1,3-glucanase	1.309	2.257	8.280E-09	-	-	-
Sugarcane_Unigene_BMK.59640	Peroxidase 16	1.641	1.457	2.940E-07	-	-	-
gi34949353	Peroxidase 4	1.587	1.683	2.210E-06	-	-	-
Sugarcane_Unigene_BMK.55512	Endo-1,4-beta-xylanase	1.991	1.918	1.110E-16	-	-	-
Sugarcane_Unigene_BMK.51436	Pathogenesis-related protein 1	1.777	1.032	4.411E-04	1.762	1.395	2.830E-08
gi36048114	Pathogenesis-related protein 1	1.290	1.552	1.420E-07	-	-	-
Sugarcane_Unigene_BMK.42413	Heat shock protein	1.287	2.000	6.440E-14	-	-	-
Sugarcane_Unigene_BMK.62025	Alcohol dehydrogenase 1	1.321	1.172	1.430E-05	-	-	-
gi6274485	pyruvate orthophosphate dikinase	1.776	1.286	3.690E-07	-	-	-
gi35938626	Expansin-B3	1.291	1.030	7.564E-04	1.256	1.132	4.420E-05
gi35030831	Beta-expansin 5	1.361	1.247	9.890E-07	-	-	-
Sugarcane_Unigene_BMK.53837	Pheophorbide a oxygenase	1.537	1.098	1.276E-04	-	-	-
Sugarcane_Unigene_BMK.57765	NADP-dependent glyceraldehyde-3-phosphate dehydrogenase	1.552	1.109	5.880E-05	-	-	-
gi35014290	1-aminocyclopropane-1-carboxylate oxidase 1	1.407	2.754	0.0000	-	-	-
gi35045219	Barwin-like Protein	4.460	1.041	7.963E-04	-	-	-
Sugarcane_Unigene_BMK.43180	Apyrase 3	1.248	1.441	5.560E-08	-	-	-
Sugarcane_Unigene_BMK.51142	5-pentadecatrienyl resorcinol O-methyltransferase	2.604	2.652	0.0000	-	-	-
Sugarcane_Unigene_BMK.61555	Fructose-bisphosphate aldolase	1.544	1.179	1.800E-05	-	-	-
Sugarcane_Unigene_BMK.66631	Primary amine oxidase	1.243	−1.208	4.28E-06	-	-	-
Sugarcane_Unigene_BMK.36357	Glycine-rich RNA-binding protein	1.413	−1.279	2.07E-06	-	-	-
Sugarcane_Unigene_BMK.66398	Protein kinase	0.722	1.283	3.35E-07	-	-	-
Sugarcane_Unigene_BMK.70494	Triacylglycerol lipase	0.825	1.073	0.000192327	-	-	-
Sugarcane_Unigene_BMK.49659	Extracellular ribonuclease	0.648	1.312	4.41E-07	-	-	-
Sugarcane_Unigene_BMK.58110	Peroxidase 2	0.734	1.239	0.000814239	-	-	-
gi35015228	Plasma membrane intrinsic protein	0.771	1.006	0.000781839	-	-	-
Sugarcane_Unigene_BMK.74968	Cellulose synthase A catalytic subunit 5	0.773	1.536	2.61E-10	-	-	-
gi35089478	Microtubule-associated protein futsch-like	0.818	1.173	0.000224862	-	-	-
Sugarcane_Unigene_BMK.50541	Peroxidase	-	-	-	1.328	1.549	2.500E-05
Sugarcane_Unigene_BMK.42924	Heat shock protein	-	-	-	2.078	1.167	4.520E-05
Sugarcane_Unigene_BMK.84564	Lectin	-	-	-	1.326	1.944	7.080E-05
Sugarcane_Unigene_BMK.48205	Glycine-rich cell wall structural protein	-	-	-	1.353	1.265	9.570E-07
Sugarcane_Unigene_BMK.44684	40S ribosomal protein S9-2	-	-	-	1.270	1.564	4.430E-11
Sugarcane_Unigene_BMK.57737	Lichenase	-	-	-	0.712	1.393	1.98E-08
Sugarcane_Unigene_BMK.73761	Acid beta-fructofuranosidase	-	-	-	0.801	1.713	1.07E-05
gi34936008	Translation elongation factor P	-	-	-	1.494	−1.093	0.000427098

Notes: Differentially expressed proteins: fold change > 1.20 (upregulated) or < 0.83 (downregulated), and *P*-value < 0.05; Differentially expressed transcripts: log_2_ fold change ≥ 1 (upregulated) or < −1 (downregulated), and FDR ≤ 0.01. YCK and YT: Yacheng05-179 under sterile water and *Sporisorium scitamineum* stresses after 48 h, respectively; RCK and RT: ROC22 under sterile water and *S. scitamineum* stresses after 48 h, respectively

**Fig. 3:**
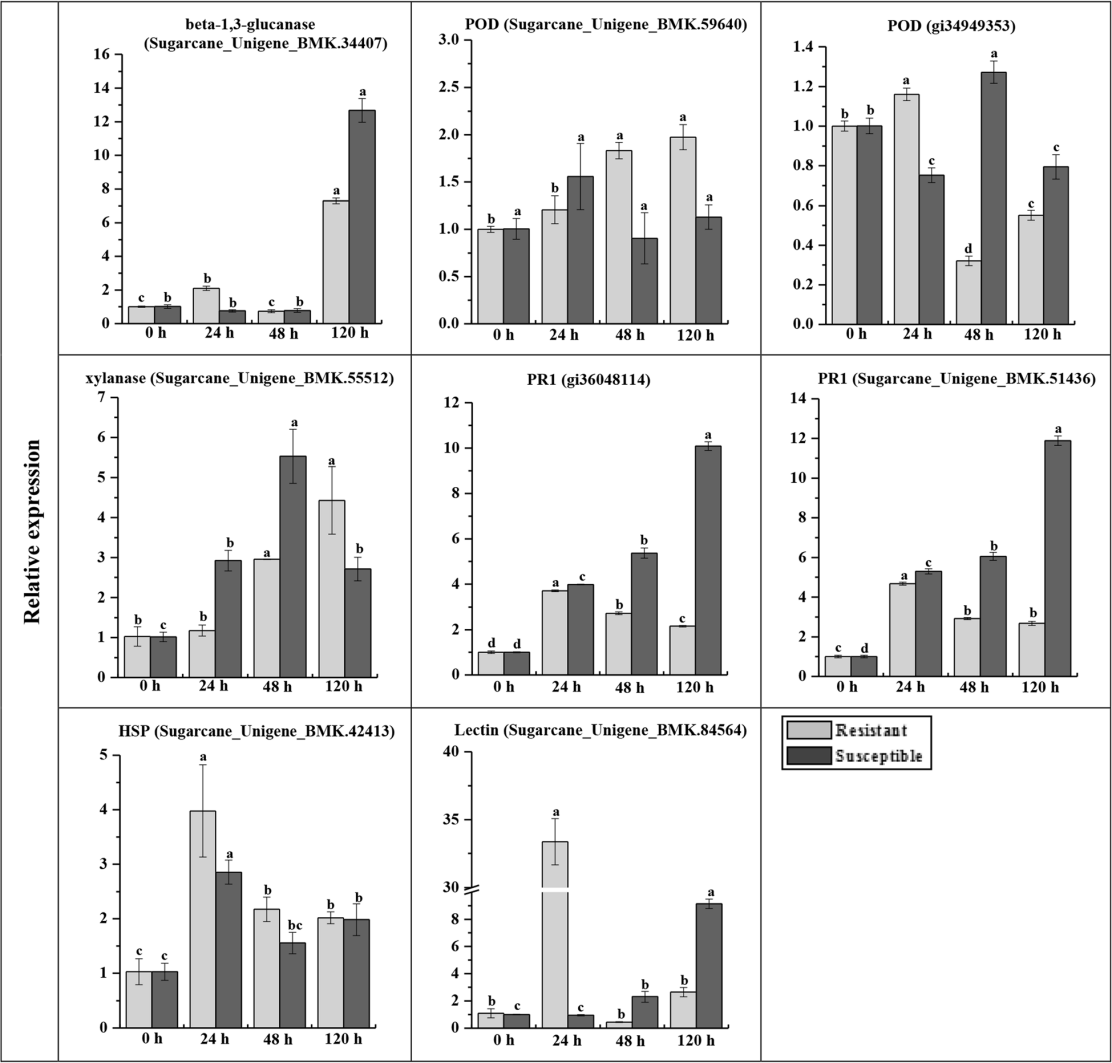
RT-qPCR analysis of parts of resistance-related differentially expressed proteins associated with the transcriptome of Yacheng05-179 and ROC22 post-*Sporisorium scitamineum* inoculation. The data of RT-qPCR were normalized to the *GAPDH* expression level. All data points are expressed as the mean ± SE (*n* = 3). Different lowercase letters indicate a significant difference, as determined by the least-significant difference test (*P*-value < 0.05). Resistant: Yacheng05-179 genotype; Susceptible: ROC22 genotype. 0, 24, 48 and 120 h, sugarcane buds inoculated with *S. scitamineum* after 0, 24, 48 and 120 h, respectively. POD, peroxidase; PR1, pathogenesis-related protein 1; HSP, heat shock protein

Candidate genes were selected for RT-qPCR analysis (Fig. 3). The gene expression profile was in agreement with our iTRAQ results. In most cases, the transcript of the target gene in Yacheng05-179 and ROC22 post *S. scitamineum* inoculation at 48 h was upregulated. In addition, the elevated amounts of SU34407, POD (gi34949353), and lectin (SU84564) transcripts were detected at as early as 24 h, and POD (SU59640) and the xylanase (SU55512) transcripts increased and lasted longer (24–120 h) in the resistant genotype compared to those of the susceptible one. These results reflected their positive contribution to *S. scitamineum* stress, and also provided the basis for the determination of the subsequent metabolic pathways and the identification of key proteins during the interaction between sugarcane and *S. scitamineum*.

### Role of SU34407 in response to pathogen infection

**Fig. 4:**
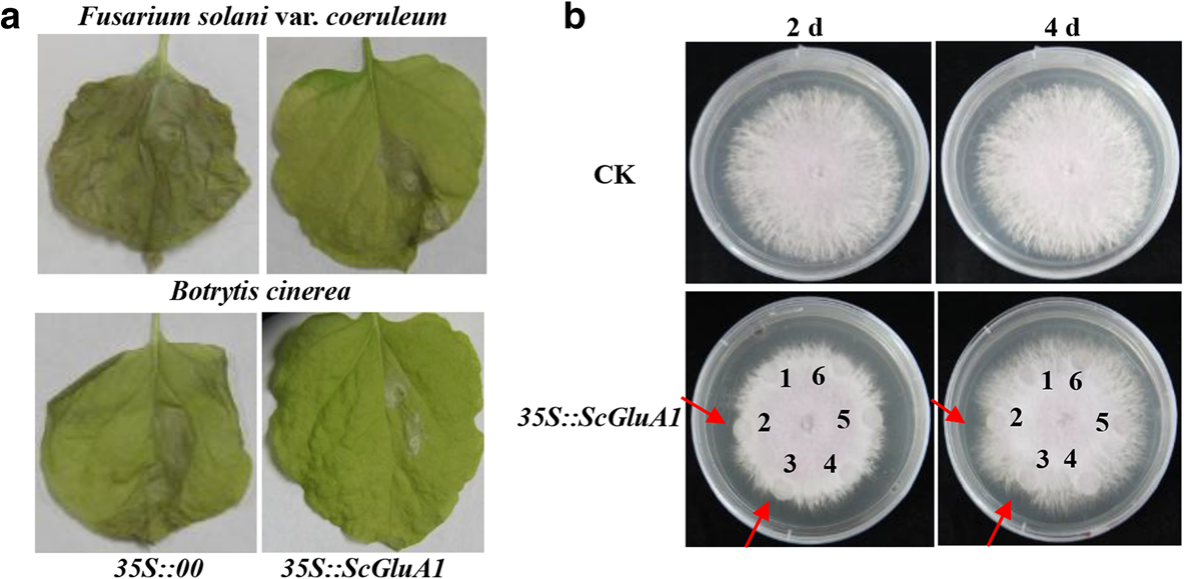
Functional analysis of the *ScGluA1* gene encoding sugarcane beta-1,3-glucanase. **a** The infection results of *Nicotiana tabacum Fusarium solani* var. *coeruleum* and *Botrytis cinerea* after infiltration with the *35S::ScGluA1*-containing *Agrobacterium* strain. Disease symptoms were assessed at 20 d post-inoculation. **b** The antimicrobial action of beta-1,3-glucanase (T_0_ generation of *ScGluA1* transgenic *N. benthamiana*) on the hyphal growth of *F. solani* var. *coeruleum.* CK, the control of normal culture on *F. solani* var. *coeruleum*; *35S::ScGluA1*, the antimicrobial action of beta-1,3-glucanase of the T_0_ generation of *ScGluA1* transgenic *N. benthamiana*; 1 ~ 3, beta-1,3-glucanase from three different T_0_ generation plants of *ScGluA1* transgenic *N. benthamiana*, respectively; 4 and 5, beta-1,3-glucanase from T_0_ generation of pCAMBIA 1301 transgenic and non-transgenic *N. benthamiana*, respectively; 6, 0.05 M sodium acetate buffer (pH 5.0). *Red arrow* indicates the antifungal effect. 2 d and 4 d, culture for 2 d and 4 d at 28 °C after crude enzyme fluid was added to the medium

In the analysis of differentially expressed proteins associated with the transcriptome, a PR protein, beta-1,3-glucanase (ScGluA1, SU34407) was observed in Yacheng05-179 post smut pathogen inoculation at 48 h at both the transcript and protein levels but remained stable in ROC22 (Table 2). To determine the antifungal characteristics of *ScGluA1*, an overexpressing vector of pCAMBIA 1301-*ScGluA1* was constructed. Approximately 20 d after inoculation with *F. solani* var. *coeruleum* or *B. cinerea* in *N. benthamiana*, the leaves of the control (*35S::00*) presented more obvious disease symptoms than those of the *35S::ScGluA1* (Fig. 4a). Furthermore, the antifungal effect *in vitro* showing hyphal growth of *F. solani* var. *coeruleum* was inhibited by the beta-1,3-glucanase enzyme from plant 2 and plant 3 of the T_0_ generation of *35S::ScGluA1* transgenic *N. benthamiana* (Fig. 4b).

### Differences in protein expression events in response to *S. scitamineum* infection in Yacheng05-179 and ROC22

Previous researches have examined pathways, genes, and transcripts involved in *S. scitamineum* resistance [13, 45]. Here, we attempted to demonstrate the role of these proteins and pathways involved in the smut pathogen resistance in Yacheng05-179 and ROC22 after challenging with *S. scitamineum*.

#### Calcium signaling pathway

**Fig. 5:**
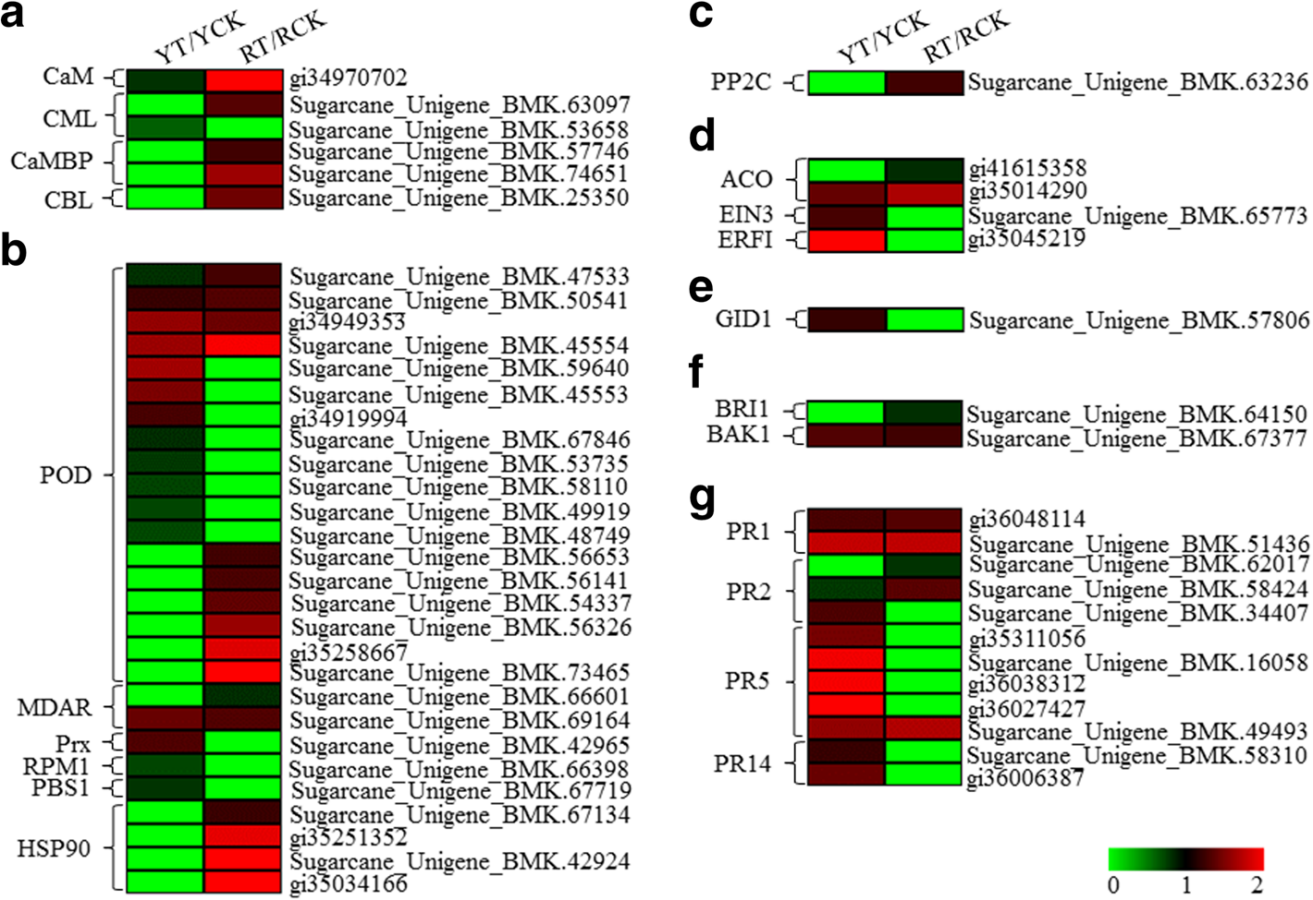
Heat maps showing the up- or downregulated proteins in specific classes in Yacheng05-179 (YT/YCK) and ROC22 (RT/RCK) post-*Sporisorium scitamineum* inoculation. The bottom *color bar* indicates the fold change value for each differentially expressed protein. Differentially expressed proteins: fold change > 1.20 (upregulated) or < 0.83 (downregulated), and *P*-value < 0.05. The fold change of the undetected value, designated as 0, is represented at the left end (*lime green*) of the colored bar. Comparisons of gene expression in YT/YCK and RT/RCK for calcium signaling pathway (**a**), ROS/NO pathway (**b**), proteins involved in the ABA pathway (**c**), ET pathway (**d**), GA pathway (**e**), BR pathway (**f**), and PRs (**g**)

Ca^2+^ is a second messenger in plant responses to pathogen attacks [46]. In the present study, none of the Ca^2+^ transporters such as Ca^2+^ ATPase was differentially expressed in Yacheng05-179 or ROC22 post-*S. scitamineum* inoculation at 48 h. However, in ROC22, several Ca^2+^ flux-related proteins were upregulated, including one calmodulin (CaM, gi34970702), one calcium-binding protein (CML, SU63097), two calmodulin-binding protein (CaMBP, SU57746 and SU74651) and one calcineurin B-like protein (CBL, SU25350), whereas in Yacheng05-179, the Ca^2+^ flux related proteins (gi34970702, SU53658 and SU25350) were downregulated or remained unchanged (SU57746, SU74651) (Fig. 5a). These result indicated that calcium signaling pathway might not play a significant role in sugarcane defense against *S. scitamineum* or even compromised smut resistance in sugarcane.

#### ROS and NO

Plant defense to stress is often regulated by ROS and NO with the induction of programmed cell death (PCD) through the establishment of a hypersensitive reaction (HR) within the infected tissue [47]. We found that the expression of 18 PODs (Fig. 5b), which are important members of the protective enzyme system in plants, was induced by *S. scitamineum* and four (SU47533, SU50541, gi34949353 and SU45554) out of these were activated in both genotypes. Among these PODs, six (SU50541, gi34949353, SU45554, SU59640, SU45553 and gi34919994) were upregulated and another six (SU47533, SU67846, SU53735, SU58110, SU49919 and SU48749) were downregulated in Yacheng05-179, whereas 10 (SU47533, SU50541, gi34949353, SU45554, SU56653, SU56141, SU54337, SU56326, gi35258667 and SU73465) were all upregulated in ROC22. Monodehyedroascorbate reductase (MDAR) is the key enzyme in the ascorbate acid (AsA) cycle [48]. Here, two differentially expressed MDAR were detected, one MDAR (SU69164) was upregulated in both sugarcane genotypes, whereas the other (SU66601) was downregulated in ROC22. Furthermore, the upregulated peroxiredoxin (Prx, SU42965) that is involved in NO only production was only detected in Yacheng05-179. These results indicated that NO only was accumulated in Yacheng05-179, whereas ROS was activated both in Yacheng05-179 and ROC22. In the HR system, one effector-triggered immune (ETI) receptor RPM1 (SU66398) and one serine-threonine kinase PBS1 (SU67719) were both downregulated in Yacheng05-179, but remained unchanged in expression in ROC22 [49, 50]. Four heat shock protein 90 (HSP90) (SU67134, gi35251352, SU42924 and gi35034166) related to HR were all upregulated in ROC22, whereas these remained stable in Yacheng05-179. These results revealed that ROS and NO might not be the key pathways for *S. scitamineum* resistance in sugarcane.

#### Phytohormones

Phytohormones play a crucial role in the responses of plants to pathogen attacks [51]. In the present study, no change in expression was observed in any of the SA and JA related proteins. However, some of the other plant hormones such as the ABA, ET, GA, and gibberellin (BR), were induced in response to *S. scitamineum* attack.

ABA has a negative effect on plant disease resistance [52]. Our analysis showed that one protein phosphatase 2C (PP2C, SU63236) that was responsible for ABA signaling was upregulated in ROC22 only, but remained unchanged in Yacheng05-179 (Fig. 5c). This implied that the ABA pathway was not involved or at least unimportant in the defense response of sugarcane to *S. scitamineum*. In contrast to ABA, GA is considered as a positive regulator of plant defense [51]. In the present study, the expression of one GA receptor GA-insensitive dwarf 1 (GID1, SU57806) was upregulated in Yacheng05-179, but remained unchanged in ROC22 (Fig. 5e), indicating that the GA pathway was only activated in a sugarcane-*S. scitamineum* incompatible reaction.

The role of ET in plant defense against pathogens has been under intense debate [46, 53]. In the present study, four proteins involved in the ET pathway were observed, including two 1-aminocyclopropane-1-carboxylate oxidases (ACOs) that were responsible for ET biosynthesis, as well as one ethylene-sensitive 3 (EIN3) and one ethylene response factor 1 (ERF1) that was responsible for ET signaling (Fig. 5d). One ACO (gi35014290) was upregulated in both sugarcane genotypes and the other one (gi41615358) was downregulated in ROC22 only, but remained unchanged in Yacheng05-179. One EIN3 (SU65773) and one ERF1 (gi35045219) were both upregulated in Yacheng05-179, whereas it remained stable in ROC22. These findings revealed that the ET biosynthesis pathway might be involved in the defense response of sugarcane to *S. scitamineum*.

BR induces disease resistance in plants [54]. Brassinosteroid insensitive 1 (BRI1) and brassinosteroid insensitive 1-associated receptor kinase 1 (BAK1) is a receptor kinase pair that mediates brassinosteroid signaling [55]. Here, one BAK1 (SU64150) was repressed in ROC22, whereas one BRI1 (SU67377) was upregulated in both genotypes (Fig. 5f). These findings demonstrated that BR was activated in both Yacheng05-179 and ROC22 after *S. scitamineum* attack.

#### Pathogenesis-related proteins

PRs such as chitinase and beta-1,3-glucanase, have been induced in sugarcane post smut pathogen inoculation [13, 56]. In the present study, among the differentially expressed proteins, 12 PRs were observed, including two PR1, three beta-1,3-glucanases (PR2), one osmotin-like protein (PR5), two thaumatin-like proteins (PR5), two zeamatin-like proteins (PR5) and two non-specific lipid-transfer proteins (PR14) (Fig. 5g). For PR1, two (gi36048114 and SU51436) were upregulated in both genotypes. For PR2, one (SU34407) was upregulated in Yacheng05-179, but remained unchanged in ROC22; another one (SU62017) was downregulated in ROC22, but remained stable in Yacheng05-179; whereas the third one (SU58424) was downregulated in Yacheng05-179, but upregulated in ROC22. Among the PR5, only one (SU49493) was upregulated in both genotypes, whereas the remaining four (gi35311056, SU16058, gi36038312 and gi36027427) were all upregulated in Yacheng05-179, but remained unchanged in ROC22. However, both two PR14 (SU58310 and gi36006387) were upregulated only in Yacheng05-179. These findings suggested that more PRs were upregulated in the resistant genotype (Yacheng05-179), which in turn might help enhance sugarcane resistance to *S. scitamineum*.

#### Phenylpropanoid metabolism

**Fig. 6:**
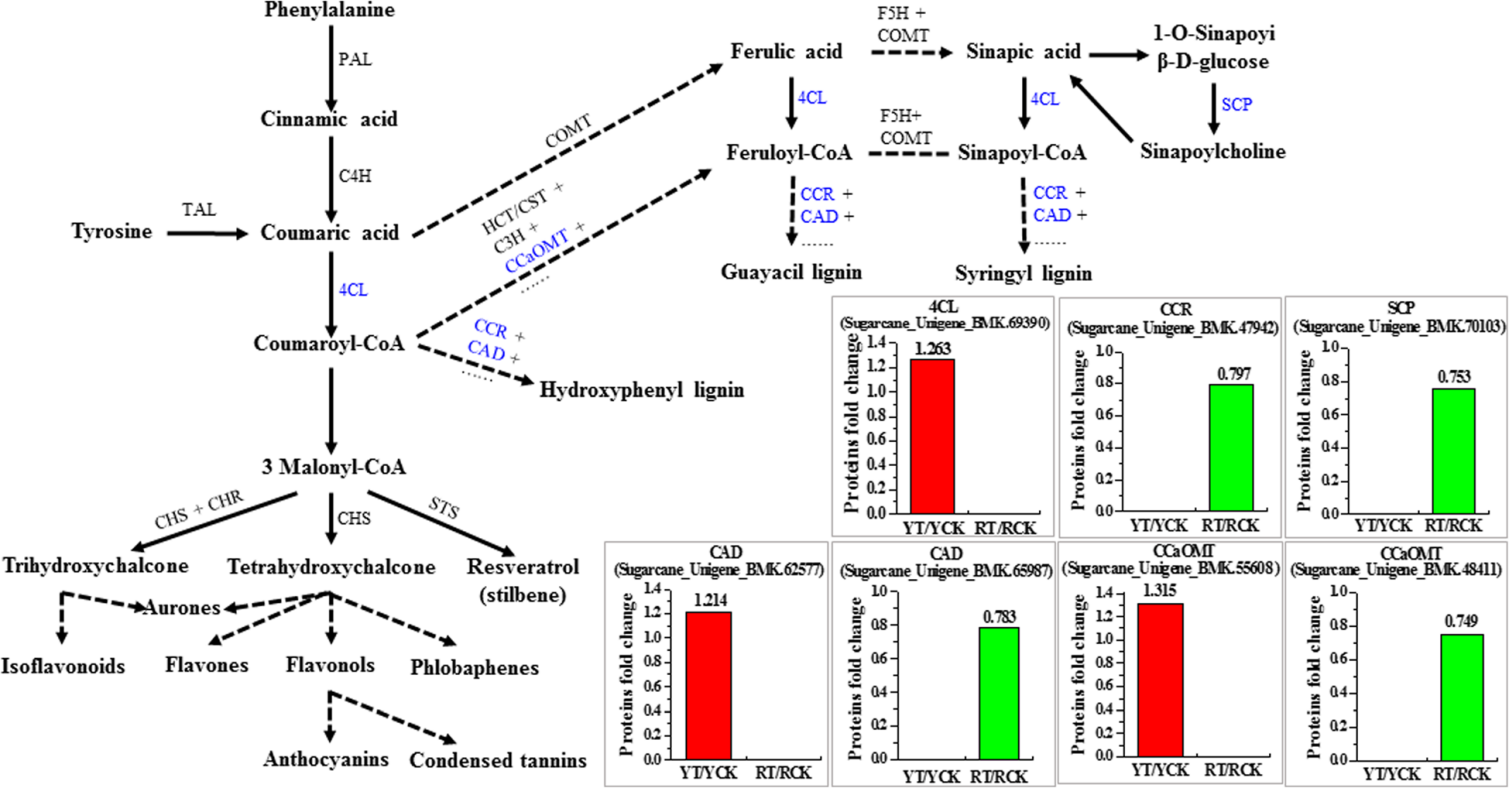
Schematic of proteome data involved in phenylpropanoid metabolism. The pathway shows two branches that lead to the production of flavonoids (*left bottom*) and lignin monomers (*right*). The solid arrow represents a single step enzymatic reaction and the dashed arrow represents multiple sequential enzymatic reactions. Blue enzymes indicate the observed differentially expressed proteins. *Red* and *green* columns represent the upregulated and downregulated proteins, respectively. YCK and YT: Yacheng05-179 under sterile water and *Sporisorium scitamineum* stresses after 48 h, respectively; RCK and RT: ROC22 under sterile water and *S. scitamineum* stresses after 48 h, respectively. TAL, tyrosine ammonia lyase; PAL, phenylalanine ammonia lyase; C4H, cinnamate 4-hydroxylase; 4CL, 4-coumarate CoA ligase; COMT, caffeic acid O-methyltransferase; HCT/CST, hydroxycinnamoyl CoA:shikimate/quinate hydroxycinnamoyl transferase; C3H, coumaroyl shikimate/quinate 3-hydroxylase; CCaOMT, caffeoyl CoA O-methyltransferase; CCR, cinnamoyl CoA reductase; CAD, cinnamyl alcohol dehydrogenase; F5H ferulate 5-hydroxylase; CHS, chalcone synthase; CHR, chalcone reductase; STS, stilbene synthase; SCP, serine carboxypeptidase

From the point of view of carbon flow, the phenylpropanoid metabolism pathway is one of the most important secondary metabolism pathways in plants [57, 58]. In the present study, KEGG enrichment analysis has revealed that the differentially expressed proteins in Yacheng05-179 were significantly (*P*-value < 0.01) involved in phenylalanine metabolism, phenylpropanoid biosynthesis, biosynthesis of secondary metabolites and benzoxazinoid biosynthesis, whereas in ROC22, these were related to fatty acid metabolism, biosynthesis of unsaturated fatty acids, phenylalanine metabolism and phenylpropanoid biosynthesis. Here, the protein changes in the phenylpropanoid biosynthesis in both sugarcane genotypes were highlighted (Fig. 6). Seven proteins, including one 4-coumarate CoA ligase (4CL), one cinnamoyl CoA reductase (CCR), two cinnamyl alcohol dehydrogenase (CAD), two caffeoyl CoA O-methyltransferase (CCaOMT), and one serine carboxypeptidase (SCP), were differentially expressed. The 4CL protein (SU69390) was upregulated only in Yacheng05-179, but remained unchanged in ROC22. One CAD (SU62577) and one CCaOMT (SU55608) were upregulated in Yacheng05-179, whereas another CAD (SU65987) and CCaOMT (SU48411) were downregulated in ROC22. In addition, one CCR (SU65773) and one SCP (gi35045219) were both downregulated in ROC22, whereas these remained stable in Yacheng05-179. The upregulation of these proteins in Yacheng05-179 revealed that phenylpropanoid metabolism might be involved in the defense response of sugarcane to *S. scitamineum*.

### Validation of iTRAQ data for selected differentially expressed proteins by MRM

**Fig. 7:**
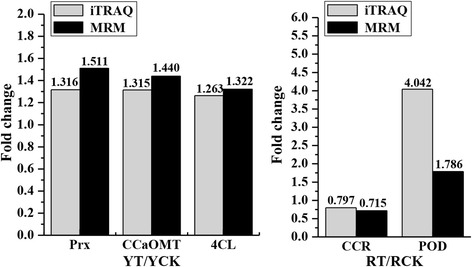
Relative expression levels of 5 selected differentially expressed proteins measured by iTRAQ and MRM in Yacheng05-179 (YT/YCK) and ROC22 (RT/RCK) post-*Sporisorium scitamineum* inoculation. Prx (SU42965), peroxiredoxin; CCaOMT (SU55608), caffeoyl CoA O-methyltransferase; 4CL (SU69390), 4-coumarate CoA ligase; CCR (SU47942), cinnamoyl CoA reductase; POD (SU73465), peroxidase. Beta-galactosidase peptides were used as internal standards in the MRM validation. Differentially expressed proteins: fold change > 1.20 (upregulated) or < 0.83 (downregulated), and *P*-value < 0.05

To verify the proteome quantitative data derived from iTRAQ, three (Prx, SU42965; CCaOMT, SU55608; 4CL, SU69390) and two (CCR, SU47942; POD, SU73465) differentially expressed proteins in Yacheng05-179 and ROC22 were selected for MRM validation, respectively. The transition for each peptide was listed in Additional file 9: Table S7. Beta-galactosidase peptide was used as internal standards. The expression trend of the five target proteins detected by MRM, including four upregualed (Prx, CCaOMT, 4CL, POD) and one (CCR) downregulated differentially expressed proteins, were consistent with those from the iTRAQ data (Fig. 7). This result demonstrated that the iTRAQ data in the present study were highly reliable for further analysis and that our definition of differentially expressed proteins was likely adequate to identify the central metabolism changes in sugarcane in response to *S. scitamineum* stimulus.

## Discussion

### Comparative proteome and transcriptome analysis of sugarcane in response to *S. scitamineum* provided novel insights into sugarcane smut resistance

Smut is a serious fungal disease that affects the sugarcane industry [1]. Due to the uncompleted sugarcane genome sequencing, transcriptome and proteome analyses are considered as an important means for elucidating the mechanism underlying disease-resistance mechanism in sugarcane [4]. To date, our understanding of the molecular mechanism underlying the defense response of sugarcane to the smut pathogen is limited, particularly at the level of proteome. In the present study, the iTRAQ method was first employed to identify proteins that are potentially involved in the sugarcane-*S. scitamineum* interaction, and 273 and 341 differentially expressed proteins were detected in Yacheng05-179 and ROC22, respectively (Fig. 1). In addition, 58 differentially expressed proteins were shared between these two genotypes. These shared and unique proteins may provide novel insights into the proteome profile involving the response of different sugarcane genotypes to *S. scitamineum* infection.

Comparative analysis showed that after infection with *S. scitamineum*, the correlation ratios between the proteome and transcriptome were 0.1502 and 0.2466 for Yacheng05-179 and ROC22, respectively (Fig. 2). The inconsistence in proteins and genes revealed that both translational and post-translational regulations play an important but diverse role in sugarcane defense against *S. scitamineum* infection. The low correlation coefficient of the proteome and transcriptome data was similar to those of previous reports [35, 59, 60]. Wu et al. [35] determined that of 130 differentially expressed proteins in the spontaneous late-ripening *Citrus sinensis* mutant and its wild type at 170 d, 190 d and 210 d after flowering, only 54 had corresponding transcripts in the RNA-seq data. In the study of transcriptomics and proteomics of *Eucalyptus* induced by *Calonectria pseudoreteaudii* for 12 h, the correlation coefficient was only 0.2935 [59]. Most differentially expressed proteins in *Eucalyptus* were involved in plant stress resistance, such as PRs, POD, chitinase, phenylalanine ammonialyase, glutathione S-transferases and dehydrins [59]. After challenging by *Candidatus liberibacter* for 50 d, root samples of Jiangxi red tangerine (*C. reticulata*) were subjected to RNA-seq and iTRAQ analysis, and 36 out of 78 differentially expressed proteins were correlated to the transcriptome data [60]. Half of these differentially expressed proteins were stress/disease resistance-related such as POD, HSP, chitinase, resistance proteins, and thaumatin-like proteins [60]. Here, 27 and 10 differentially expressed proteins associated with the transcriptome were detected in the smut-resistant and susceptible genotypes, respectively. In addition, the number of the differentially expressed proteins, which were expressed at a similar upregulated trend to that of differentially expressed genes, was 18 and 10, respectively. Most differentially expressed proteins were also closely related to plant stress or disease resistance, for instance, PODs, PRs, beta-1,3-glucanase, HSP, and lectin (Table 2). These results provide an overview of the protein expression profile of sugarcane after *S. scitamineum* attack and may be utilized in the identification of candidate resistance proteins in sugarcane breeding of smut resistance.

### ET, GA, and phenylpropanoid metabolism pathways play positive role in smut resistance in sugarcane

A previous study has shown that SA or JA/ET plays an essential role in plant disease response [61]. SA is important but not the indispensable mobile signal to the establishment of systemic acquired resistance (SAR) [62]. Loake and Grant [62] determined that SAR is associated with plant defense to biotrophic and hemi-biotrophic pathogens. On the contrary, JA and ET can synergistically activate defense responses against necrotrophic pathogens and herbivorous insects [63]. However, in the present study, none of SA- and JA-related proteins were detected at 48 h post *S. scitamineum* inoculation in smut resistant or susceptible sugarcane genotypes. This may be due to the late sampling time point selection during the interactive stage of *S. scitamineum* infection or its low expression abundance immediately after inoculation, which was in accordance with the previous report that JA has an earlier role in establishing SAR [64].

**Fig. 8:**
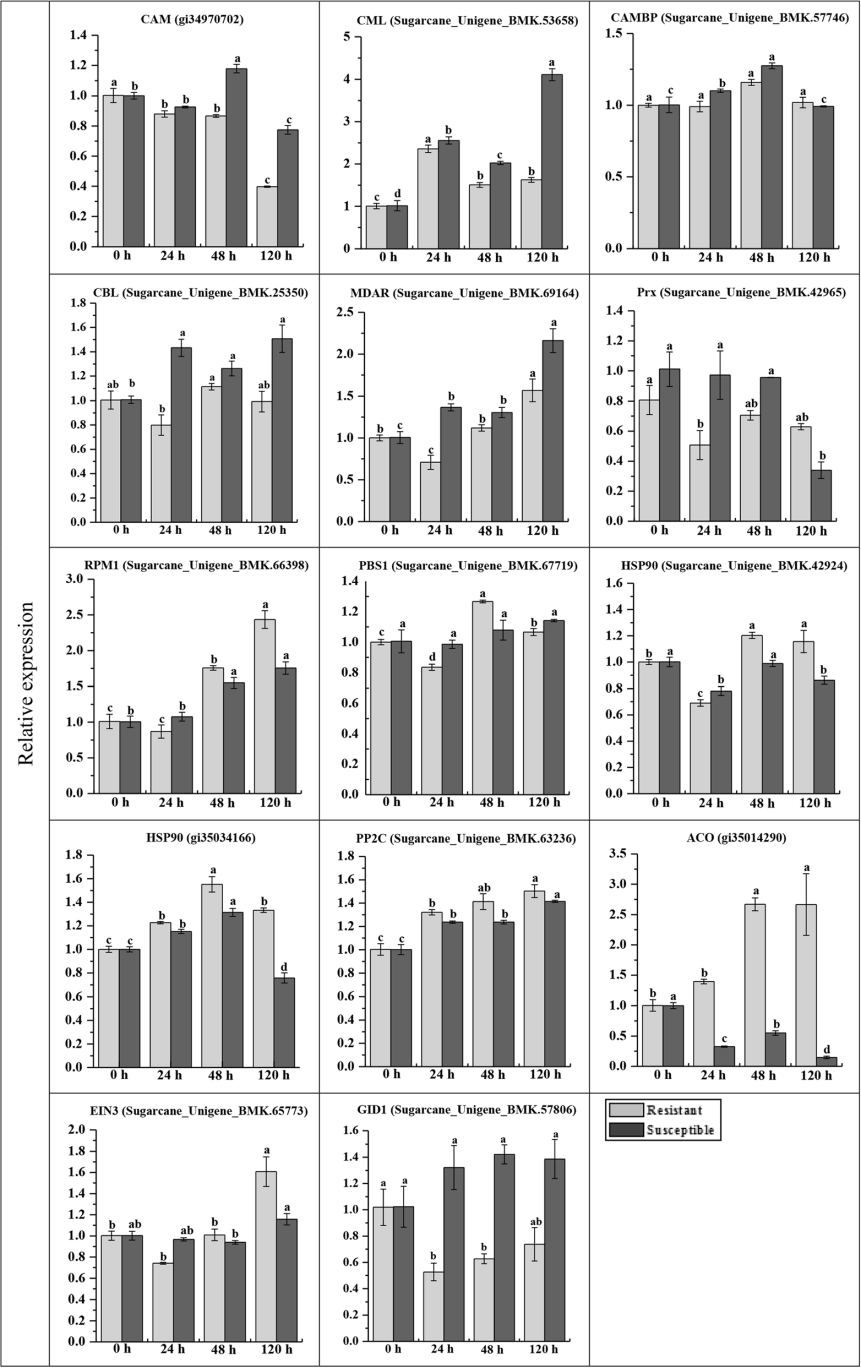
Expression profiles of differentially expressed proteins involved in the calcium, ROS/NO, ABA, ET and GA pathways in Yacheng05-179 and ROC22 post-*Sporisorium scitamineum* inoculation. The data of RT-qPCR were normalized to the *GAPDH* expression level. All data points are expressed as the mean ± SE (*n* = 3). Y, Yacheng05-179; R, ROC22. 0, 24, 48, and 120 h, sugarcane buds inoculated with *S. scitamineum* at time points of 0, 24, 48, and 120 h, respectively. CaM, calmodulin; CML, calcium-binding protein; CaMBP, calmodulin-binding protein; CBL, calcineurin B-like protein; MDAR, monodehyedroascorbate reductase; Prx, peroxiredoxin; RPM1, effector-triggered immune receptor; PBS1, serine-threonine kinase; HSP90, heat shock protein 90; PP2C, protein phosphatase 2C; ACO, 1-aminocyclopropane-1-carboxylate acid oxidase; EIN3, ethylene sensitive 3; and GID1, GA-insensitive dwarf 1

ET is a principal modulator in plant-pathogen interaction [65]. For the ET pathway, two categories of proteins including ET biosynthesis and ET signaling were induced in Yacheng05-179. The expression patterns of the ET pathway-related genes such as *ACO* (gi35014290) and *EIN3* (SU65773) were verified by RT-qPCR (Fig. 8). Ding et al. [66] reported that the ACO enzyme that is responsible in catalyzing 1-aminocyclopropane-1-carboxylate acid (ACC) to ET was only induced in the resistant *T. aestivum* genotype infected by *F. graminearum*. In the present study, one out of two ACO (gi35014290) was upregulated in both sugarcane genotypes. However, the expression pattern of *ACO* gene was upregulated and maintained longer (48 h–120 h) in the resistant genotype, but downregulated in the susceptible one (Fig. 8), which indicated its positive effect on the sugarcane smut resistance. Lorenzo et al. [67] revealed that the transcription factor ERF1 has a positive effect on ET and JA signaling. Several ERF family members could modulate plant defense gene expression and disease resistance [68]. EIN3 is a positive component in activating the ET pathway [69, 70]. Here, EIN3 (SU65773) and ERF1 (gi35045219) were responsive to the smut pathogen attack in Yacheng05-179, but not ROC22. These results suggested that ET pathway might be associated with *S. scitamineum* resistance in sugarcane.

GA is identified as a signaling factor in plant response to pathogen attacks [53]. Xin et al. [53] have demonstrated that ent-kaurene synthase and ent-kaurene oxidase in the GA biosynthesis pathway are upregulated in *T. aestivum* after *Blumeria graminis* f. sp. *tritici* inoculation. Bari and Jones [51] have reported that the resistance of *Arabidopsis* to *P. syringae* pv. *tomato* DC3000 increased after the addition of exogenous GA. In the present study, the GA pathway responded to the *S. scitamineum* infection in Yacheng05-179 via the upregulation of the GID1 protein at 48 h, whereas no expression in ROC22 was observed (Fig. 5e). In Fig. 8, the gene expression pattern of *GID1* was repressed in Yacheng05-179 and remained stable in ROC22. The difference in the expression levels of *GID1* in Yacheng05-179 between iTRAQ and RT-qPCR analysis could be attributed to the regulatory mechanisms at the transcriptional and post-transcriptional levels in sugarcane after the *S. scitamineum* attack. This was similar to the findings of Tanaka et al. [71] and Fan et al. [72].

Phenylpropanoids such as lignin, flavonoids, coumarins, and phenolic compounds play vital roles in the defense of plants against pathogen attack [73]. They take part in multiple branches of the phenylpropanoid metabolism pathway [58]. In the present study, 4CL, which plays key role in the phenylpropanoid metabolism pathway and responds to various biotic and abiotic stresses [59], was upregulated in Yacheng05-179. Lignin is essential for defense against pathogens as it presents an undegradable mechanical barrier to most pathogens [74, 75]. In plants, there are three different types of lignin, including hydroxyphenyl (H), guaiacyl (G), and syringyl (S) (Fig. 6). Our analysis indicated that seven proteins involved in lignin biosynthetic pathway were induced by *S. scitamineum*, including 4CL, CCR, CAD, CCaOMT, and SCP. Interestingly, three of these (SU69390, SU62577, and SU55608) were all upregulated in the resistant genotype but remained unchanged in the susceptible one, whereas the other four (SU65987, gi35045219, SU48411 and SU65773) were all downregulated in the susceptible genotype but remained stable in the resistant one. CCR and CAD, the first two enzymes required for monolignol synthesis, are involved in defense signaling during pathogen infection [76]. In *O. sativa*, *OsCCR1* plays an important role in defense responses, including a role in the production of ROS, which is mediated by nicotinamide adenine dinucleotide phosphate (NADPH) oxidase [75]. The specific activation of an S lignin biosynthesis pathway in *T. aestivum* and its relationship to the deposition of a defense was induced by CAD [77]. CCaOMT, one of the key enzymes in lignin biosynthesis pathway, is crucial to the formation of G and S lignin [78]. The upregulation of *CCaOMT* in plants leads to an increase in lignin content [79]. SCPs, as a class of proteases belonging to the hydrolase family, are involved in various aspects of peptides and proteins that modify and disrupt plant growth and development [80]. Moreover, they play an important role in various biochemical pathways, including secondary metabolite biosynthesis. Liu et al. [81] showed that the expression of *OsBISCPL1* was significantly upregulated in incompatible interactions between *O. sativa* and the blast fungus (*Magnaporthe grisea*). Furthermore, the *OsBISCPL1*-overexpressing plants showed enhanced disease resistance against *P. syringae* pv. *tomato* and *Alternaria brassicicola* [81]. Therefore, we can speculate that the phenylpropanoid metabolism pathway in sugarcane may be associated with the accumulation of secondary metabolites in response to *S. scitamineum* infection.

### Calcium signaling, ROS/NO, and ABA pathways are not essential to smut resistance or do not influence smut resistance in sugarcane

Calcium is a major signal molecule in plant signal transduction cascades [82]. Ca^2+^ influx serves as an early signal of plant disease resistance response to pathogen perception [83]. CaM, the most important Ca^2+^ sensor, can adjust the physiological function of cells by interacting with downstream CaMBP [84]. CML and CBL serve as Ca^2+^-binding proteins in plant cells [84]. In the present study, we demonstrated that the expression of the CaM, CML, CaMBP and CBL were all upregulated in ROC22, suggesting that these were more active than those in Yacheng05-179 after *S. scitamineum* infection. Likewise, Ca^2+^ flux-related genes, including *CAM* (gi34970702), *CML* (SU53658), *CAMBP* (SU57746) and *CBL* (SU25350), were all upregulated in ROC22 compared to those in Yacheng05-179 (Fig. 8). These findings suggest that the calcium signaling pathway may be repressed in Yacheng05-179 during *S. scitamineum* resistance. Interestingly, this finding is consistent with those of the findings reported by Xiao et al. [46], in which the calcium pathway is responsive to *F. graminearum* infection in the susceptible wheat genotype, whereas not activated in the resistant one.

ROS and NO are needed to induce HR-mediated PCD [47]. As reported, the accumulation of ROS in plants after a pathogen attack could cause tissue necrosis and increase susceptibility to necrotrophic pathogens [59], whereas it could induce resistance to biotrophic pathogens [85]. In the present study, *S. scitamineum*-infected sugarcane showed changes in the abundances of ROS scavenging (POD and MDAR) and NO producing (MDAR)-related proteins. In the HR system, the expression trends of RPM1 (SU66398), PBS1 (SU67719), and HSP90 (SU42924 and gi35034166) between iTRAQ (Fig. 5b) and RT-qPCR analyses were opposite (Fig. 8), suggesting the HR system was established in both sugarcane genotypes. However, Xiao et al. [46] have indicated that Ca^2+^ influx can induce oxidative burst and in turn, the generation of ROS induces an influx of Ca^2+^. Similar to the findings on calcium signaling pathway [46], the upregulation of the POD superfamily protein, MDAR, and HSP90 in ROC22 indicated that the ROS/NO as well as PCD/HR were more active in ROC22 than in Yacheng05-179 during sugarcane-*S. scitamineum* interaction for 48 h.

The ABA pathway has been reported to be a negative regulator of plant disease resistance [51, 52]. Compared to wild-type plants, ABA-deficient *Arabidopsis* mutants *aba1-1* and *aba2-1* were more resistance to *Hyaloperonospora arabidopsidis* and *F. oxysporum*, respectively [86, 87]. Also, after ABA application, the plant-specific resistance reactions of *O. sativa* to *M. grisea* [88] and *Arabidopsis* to *P. syringae* pv. *tomato* [89] were suppressed. PYR/PYL/RCAR (pyrabatin resistance/pyrabatin resistance 1-like/regularoly component of ABA receptors)-|PP2C-|SnRK2 (sucrose nonfermenting-1-related protein kinase 2) comprise a double-negative regulatory system that adjusts the ABA signaling and reaction of downstream genes [13]. Rodriguez [90] reported that the overexpression of *Arabidopsis AthPP2CA* blocks ABA-inducible gene expression. In the present study, one PP2C protein (SU63236) was upregulated in ROC22 only, which was in accordance with a previous report that identified it as a negative regulator of ABA response [91]. This result was also similar to the findings of our previous report that three, two and one *PP2C* transcripts were downregulated in Yacheng05-179 at 24, 48, and 120 h post-inoculation with *S. scitamineum*, respectively [13]. Meanwhile, in Fig. 8, the expression of the *PP2C* gene (SU63236) was upregulated at a higher level in Yacheng05-179 than that in ROC22. Therefore, we deduced that the ABA pathway might not be related to smut resistance response or even compromised smut resistance in sugarcane.

### The positive responses of PRs contribute to sugarcane resistance to *S. scitamineum* attack

PRs play an important role in plant disease resistance and are closely related to SAR [92]. In the present study, we observed that several PRs were differentially expressed during sugarcane-*S. scitamineum* interactions, including two PR1, three PR2, five PR5 and two PR14 (Fig. 5g). In addition, a higher accumulation of PRs was detected in Yacheng05-179 than in ROC22. The beta-1,3-glucanase ScGluA1 (SU34407) was upregulated at both the transcript and protein levels in Yacheng05-179, which in turn warrants an investigation on the role of its encoding gene. Besides, the overexpression of *ScGluA1* in *N. benthamiana* showed an antimicrobial action on *F. solani* var. *coeruleum* and *B. cinerea* (Fig. 4a). Moreover, beta-1,3-glucanase enzyme from the T_0_ generation of *ScGluA1* transgenic *N. benthamiana* inhibited the hyphal growth of *F. solani* var. *coeruleum* (Fig. 4b). Similarly, Boggs and Jackson [93] indicated that beta-1,3-glucanase from *Arthrobacter* spp. inhibits the germination of *Bremia lactucae*in vitro. Therefore, beta-1,3-glucanase may be a component of the sugarcane defense mechanisms against *S. scitamineum*, which was in accordance with the result reported by Gu et al. [94]. Further extensive work is necessary to define the roles of many other proteins in the smut resistant process.

## Conclusions

**Fig. 9:**
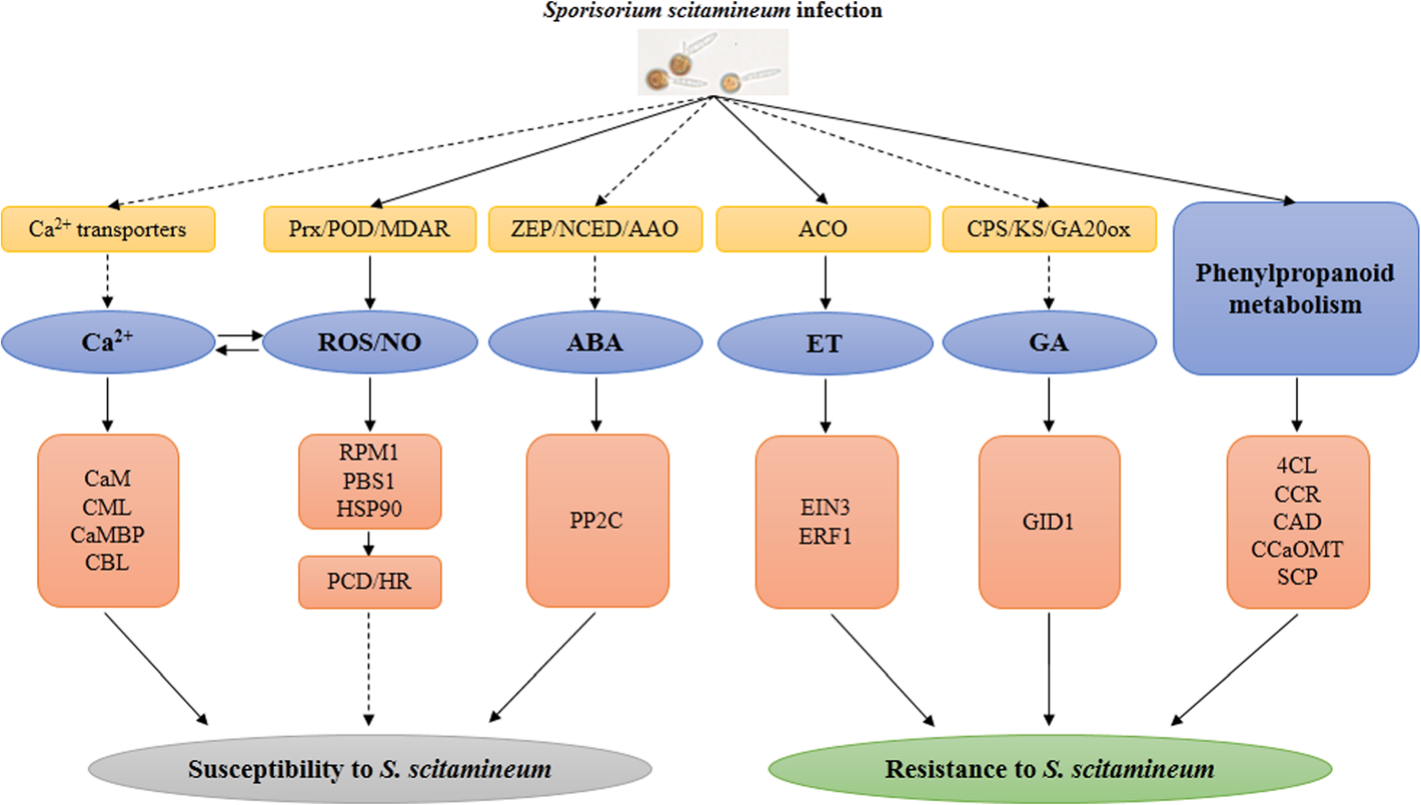
A proposed working model for the calcium, ROS/NO, ABA, ET, GA and phenylpropanoid metabolism pathways in the regulation of sugarcane resistance or susceptibility to *Sporisorium scitamineum*. The dashed arrow represents the potential roles of those pathways in the response to the smut pathogen in sugarcane. Ca^2+^, calcium; CaM, calmodulin; CML, calcium-binding protein; CaMBP, calmodulin-binding protein; CBL, calcineurin B-like protein; ROS, reactive oxygen species; NO, nitric oxide; Prx, peroxiredoxin; POD, peroxidase; MDAR, monodehyedroascorbate reductase; RPM1, effector-triggered immune receptor; PBS1, serine-threonine kinase; HSP90, heat shock protein 90; PCD, programmed cell death; HR, hypersensitive reaction; ABA, abscisic acid; ZEP, zeaxanthin epoxidase; NCED, 9-cis-epoxycarotenoid dioxygenase; AAO, ABA abscisic acid; PP2C, protein phosphatase 2C; ET, ethylene; ACO, 1-aminocyclopropane-1-carboxylate acid oxidase; EIN3, ethylene sensitive 3; ERF1, ethylene response factor 1; GA, gibberellic acid; CPS, copalyl pyrophosphate synthase; KS, ent-kaurene synthase; GA20ox, GA-20 oxidase; GID1, GA-insensitive dwarf 1; 4CL, 4-coumarate CoA ligase; CCR, cinnamoyl CoA reductase; CAD, cinnamyl alcohol dehydrogenase; CCaOMT, caffeoyl CoA O-methyltransferase; SCP, serine carboxypeptidase

In the present study, an overview of the protein expression profile in sugarcane resistant (Yacheng05-179) and susceptible (ROC22) genotypes in response to *S. scitamineum* challenge at 48 h was first obtained by using the iTRAQ technique. Also, an integrated analysis showed a poor correlation between proteomics and transcriptomics, whereas most associated proteins were closely related to plant stress resistance. In addition, a putative network (Fig. 9) in the regulation of resistance of sugarcane to *S. scitamineum* was proposed. The ET, GA, and phenylpropanoid metabolism pathways as well as PRs, such as PR1, PR2, PR5 and PR14, which were more active in Yacheng05-179, might contribute to smut resistance in sugarcane. The calcium signaling, ROS/NO and ABA pathways, which were repressed by *S. scitamineum*, might not be important for smut resistance in sugarcane. These findings shed new light on the differential expression of proteins in sugarcane in response to *S. scitamineum* infection.

## Additional files

Additional file 1: Text S1. Details of the transitions selection and MRM method validation. (DOCX 16 kb)
Additional file 2: Table S1. The primers used for RT-qPCR amplification of correlated differentially expressed genes. (DOCX 23 kb)
Additional file 3: Figure S1. The detection of gDNA PCR and total RNA RT-PCR of resistance plants for *ScGluA1* gene. M, DNA marker 15,000 + 2000 bp; 1 ~ 5, The gDNA PCR amplification products of transgenic plants; 1’ ~ 5’, The total RNA RT-PCR amplification products of transgenic plants; 6 and 6’, The amplification products of pCAMBIA 1301-*ScGluA1*; 7 and 7’, The amplification products of non-transgenic plants; 8 and 8’, Blank control. (DOCX 242 kb)
Additional file 4: Table S2. List of the total 4251 proteins identified in this study. Changes in protein abundance with *P*-value less than 0.05, which was automatically executed by the Mascot 2.3.02 software, was marked up with asterisk. (XLS 5598 kb)
Additional file 5: Table S3. List of upregulated proteins identified and quantified by the iTRAQ analysis in YT/YCK. (XLS 196 kb)
Additional file 6: Table S4. List of downregulated proteins identified and quantified by the iTRAQ analysis in YT/YCK. (XLS 136 kb)
Additional file 7: Table S5. List of upregulated proteins identified and quantified by the iTRAQ analysis in RT/RCK. (XLS 263 kb)
Additional file 8: Table S6. List of downregulated proteins identified and quantified by the iTRAQ analysis in RT/RCK. (XLS 171 kb)
Additional file 9: Table S7. Transitions of five differentially expressed proteins selected for the MRM validation. (DOCX 20 kb)

## Abbreviations

1DE: One-dimensional gradient polyacrylamide gels
4CL: 4-coumarate CoA ligase
ABA: Abscisic acid
ACC: 1-aminocyclopropane-1-carboxylate acid
ACOs: 1-aminocyclopropane-1-carboxylate oxidases
ANOVA: Analysis of Variance
AsA: Ascorbate acid
BAK1: Brassinosteroid insensitive 1-associated receptor kinase 1
BR: Gibberellin
BRI1: Brassinosteroid insensitive 1
BSA: Bovine serum albumin
CAD: Cinnamyl alcohol dehydrogenase
CaM: Calmodulin
CaMBP: Calmodulin-binding protein
CBL: Calcineurin B-like protein
CCaOMT: Caffeoyl CoA O-methyltransferase
CCR: Cinnamoyl CoA reductase
cDNA-AFLP: cDNA-amplified fragment length polymorphism
CML: Calcium-binding protein
DPS: Data Processing System
EIN3: Ethylene-sensitive 3
ERF1: Ethylene response factor 1
ET: Ethylene
ETI: Effector-triggered immune
FDR: False discovery rate
GA: Gibberellic acid
GAPDH: Glyceraldehyde-3-phosphate dehydrogenase
GID1: GA-insensitive dwarf 1
GO: Gene Ontology
HPLC: High performance liquid chromatography
HR: Hypersensitive reaction
HSP: Heat shock protein
HSP90: Heat shock protein 90
IDA: Information-dependent acquisition
iTRAQ: Isobaric tags for relative and absolute quantitation
KEGG: Kyoto Encyclopedia of Genes and Genomes
LC-ESI-MS/MS: Liquid chromatography-electrospray in trap tandem mass spectrometry
MALDI-TOF-TOF/MS: Matrix-assisted laser desorption/ionization time of flight mass spectrometry
MDAR: Monodehyedroascorbate reductase
MgCl_2_: Magnesium chloride
MGF: Mascot generic format
MRM: Multiple reaction monitoring
MS: Mass spectrometry
NADPH: Nicotinamide adenine dinucleotide phosphate
NO: Nitric oxide
PBS1: Serine-threonine kinase
PCD: Programmed cell death
PDA: Potato dextrose agar
POD: Peroxidase
PP2C: Protein phosphatase 2C
PR: Pathogenesis-related protein
Prx: Peroxiredoxin
PYR/PYL/RCAR: Pyrabatin resistance/pyrabatin resistance 1-like/regularoly component of ABA receptors
RNA-seq: RNA sequencing
ROS: Reactive oxygen species
RPM1: Effector-triggered immune receptor
RT-qPCR: Reverse transcription quantitative real-time polymerase chain reaction
SAR: Systemic acquired resistance
SCP: Serine carboxypeptidase
SCX: Strong cation exchange
SE: Standard error
SnRK2: Sucrose nonfermenting-1-related protein kinase 2
TCA: Trichloroacetic acid
TEAB: Tetraethyl-ammonium bromide
TMV: Tobacco mosaic virus
