# Supplementary material for: Comparative proteomics reveals that central metabolism changes are associated with resistance against Sporisorium scitamineum in sugarcane
Source: BMC Genomics. 2016 Oct 12;17:800. doi: 10.1186/s12864-016-3146-8 (PMC5062822; doi:10.1186/s12864-016-3146-8)
Supplement: Additional file 1: Text S1. — Details of the transitions selection and MRM method validation. (DOCX 16 kb) [file 12864_2016_3146_MOESM1_ESM.docx]

**Comparative proteomics reveals that central metabolism changes are associated with resistance against *Sporisorium scitamineum* in sugarcane**

**Yachun** **Su^1^**

**E-mail:** **[syc2009mail@163.com](mailto:syc2009mail@163.com)**

**Liping Xu^1^***

**E-mail:** [**xlpmail@126.com**](mailto:xlpmail@126.com)

**Zhuqing Wang^1^**

**E-mail:** [**zhuqingemail@163.com**](mailto:zhuqingemail@163.com)

**Qiong Peng^1^**

**E-mail:** [**pengqiongfj@163.com**](mailto:pengqiongfj@163.com)

**Yuting Yang^1^**

**E-mail:** **[yytjiayou@126.com](mailto:yytjiayou@126.com)**

**Yun** **Chen^1^**

**E-mail:** **[sweetchenyun@163.com](mailto:sweetchenyun@163.com)**

**Youxiong Que^1,2^***

**E-mail:** [**queyouxiong@126.com**](mailto:queyouxiong@126.com)

^1^Key Laboratory of Sugarcane Biology and Genetic Breeding, Ministry of Agriculture, Fujian Agriculture and Forestry University, Fuzhou 350002, China

^2^Guangxi Collaborative Innovation Center of Sugarcane Industry, Guangxi University, Nanning 530005, China

***Correspondence should be addressed to** [xlpmail@126.com](mailto:xlpmail@126.com) and [queyouxiong@126.com](mailto:queyouxiong@126.com)

**The full postal address of the submitting author Youxiong Que is as follows:** Key Laboratory of Sugarcane Biology and Genetic Breeding, Ministry of Agriculture, Fujian Agriculture and Forestry University, Fuzhou 350002, China

**Additional file 1: Text S1** Details of the transitions selection and MRM method validation

**Protein extraction and digestion**

Protein extraction was carried out according to the protocol that integrated trichloroacetic acid (TCA)/acetone precipitation with a methanol wash and phenol extraction. Total protein (100 μg) was taken out of each sample solution and digested with Trypsin Gold (Promega, Madison, WI, USA) with the ratio of protein:trypsin =30:1 at 37 °C for 16 h. Then the peptides were dried by vacuum centrifugation and reconstituted in 0.5 mol tetraethyl-ammonium bromide (TEAB, Applied Biosystems, Milan, Italy).

**LC-MRM-MS**

Samples were spiked with 50 fmol of β-galactosidase for data normalization. MRM analyses were performed on a QTRAP 5500 mass spectrometer (AB SCIEX, Foster City, CA) equipped with a LC-20AD nano HPLC system (Shimadzu, Kyoto, Japan). The mobile phase consisted of 0.1 % aqueous formic acid (solvent A) and 98 % acetonitrile with 0.1 % formic acid (solvent B). Peptides were separated on a BEH130 C18 column (0.075×150 mm column, 3.6 μm; Waters) at 300 nL/min, and eluted with a gradient of 5 %−30 % solvent B for 38 min, 30 %−80 % solvent B for 4 min, and maintenance at 80 % for 8 min. For the QTRAP 5500 mass spectrometer, spray voltage of 2400 V, nebulizer gas of 23 p.s.i., and a dwell time of 10 ms were used. Multiple MRM transitions were monitored using a unit resolution in both Q1 and Q3 quadrupoles to maximize specificity.

**Transitions selection**

A spectral library of MS/MS data was generated on a TripleTOF5600 (AB SCIEX, Foster City, CA) and searched using Mascot v2.3 (Matrix Science, UK) against with a *Saccharum* database (72441 entries). The dat file was imported into Skyline software where a library was built. The peptides was selected for MRM method development according to the following criteria: (1) the peptides with unique sequence in the database; (2) a maximum m/z of peptide <1250 (limitation of Quandrupole scan), with a peptide length range 5-40 aa; (3) without Methionine in peptides; (4) with Carbamidomethyl on Cysteine and without variable modification in peptides; and (5) no missed cleavage of trypsin. We initially monitored 6 transitions per peptide to ensure specificity with the criteria that >5 y-ions with the same elution profile and in the same ratios as the spectral library. The predicted retention time of targeted peptides was observed with an iRT strategy. A pooled peptides digested as described was performed preliminary SRM assays used to determine where these proteins were detected.

**MRM method validation**

The chromatograms of all transitions generated on QTRAQP5500 were input to Skyline. MRM method of a given protein was successfully developed only if the protein had at least one unique peptide which (1) was identified with MS/MS spectral library (cut-off score >0.95), (2) had >5 fragment ions with the same elution profile and in the same ratios as the spectral library, and (3) had an accurate retention time (less than ±2 minutes deviation against to predicted retention time).
