# Supplementary material for: Comparative proteomics reveals that central metabolism changes are associated with resistance against Sporisorium scitamineum in sugarcane
Source: BMC Genomics. 2016 Oct 12;17:800. doi: 10.1186/s12864-016-3146-8 (PMC5062822; doi:10.1186/s12864-016-3146-8)
Supplement: Additional file 2: Table S1. — The primers used for RT-qPCR amplification of correlated differentially expressed genes. (DOCX 23 kb) [file 12864_2016_3146_MOESM2_ESM.docx]

**Comparative proteomics reveals that central metabolism changes are associated with resistance against *Sporisorium scitamineum* in sugarcane**

**Yachun** **Su^1^**

**E-mail:** **[syc2009mail@163.com](mailto:syc2009mail@163.com)**

**Liping Xu^1^***

**E-mail:** [**xlpmail@126.com**](mailto:xlpmail@126.com)

**Zhuqing Wang^1^**

**E-mail:** [**zhuqingemail@163.com**](mailto:zhuqingemail@163.com)

**Qiong Peng^1^**

**E-mail:** [**pengqiongfj@163.com**](mailto:pengqiongfj@163.com)

**Yuting Yang^1^**

**E-mail:** **[yytjiayou@126.com](mailto:yytjiayou@126.com)**

**Yun** **Chen^1^**

**E-mail:** **[sweetchenyun@163.com](mailto:sweetchenyun@163.com)**

**Youxiong Que^1,2^***

**E-mail:** [**queyouxiong@126.com**](mailto:queyouxiong@126.com)

^1^Key Laboratory of Sugarcane Biology and Genetic Breeding, Ministry of Agriculture, Fujian Agriculture and Forestry University, Fuzhou 350002, China

^2^Guangxi Collaborative Innovation Center of Sugarcane Industry, Guangxi University, Nanning 530005, China

***Correspondence should be addressed to** [xlpmail@126.com](mailto:xlpmail@126.com) and [queyouxiong@126.com](mailto:queyouxiong@126.com)

**The full postal address of the submitting author Youxiong Que is as follows:** Key Laboratory of Sugarcane Biology and Genetic Breeding, Ministry of Agriculture, Fujian Agriculture and Forestry University, Fuzhou 350002, China

**Additional file 2: Table S1** The primers used for RT-qPCR amplification of correlated differentially expressed genes

| **Gene Name** | **ID name** | **Forward primer (5’-3’)** | **Reverse primer (5’-3’)** |
| --- | --- | --- | --- |
| *beta-1,3-glucanase* | Sugarcane_Unigene_BMK.34407 | CCATGCTTGCACTGGCATTGCTT | TTGTCGCCGTTCACGCCGTAG |
| *POD* | Sugarcane_Unigene_BMK.59640 | CAGAGCGTGGTCGTCATAG | GAAGCAGTCGTGGAAGAAGAG |
| *POD* | gi34949353 | CATCCTCCGCCTCTTCTT | CATCTTCTCGCCCTGGAA |
| *xylanase* | Sugarcane_Unigene_BMK.55512 | ACGATTACTCCGCCTACAC | TGGTGGTCTTGTTGAGGTT |
| *PR1* | gi36048114 | GTCAGGCAGTTCAACTTCAC | TGTCCAGGTCCAGGAAATC |
| *PR1* | Sugarcane_Unigene_BMK.51436 | CAGCATCAGGCAGTTCAAC | TGTCCAGGTCCAGGAAATC |
| *HSP* | Sugarcane_Unigene_BMK.42413 | CGCAAGCACAAGAAGGAC | GGGTGATGGTGGTGTAGAA |
| *Lectin* | Sugarcane_Unigene_BMK.84564 | CCAACTGGTCCGTGTCATA | GTGGAGTCTCGTCTTCATCA |
| *CaM* | gi34970702 | AAGATGAAGGACACCGACTC | CAGCCGAGATGAAACCATTC |
| *CML* | Sugarcane_Unigene_BMK.53658 | CATTCCGCATTATTGACCAAGA | GCTAGTCCTCCTCATCATCC |
| *CaMBP* | Sugarcane_Unigene_BMK.57746 | TGGTGAATGATGTGGAAGAGA | CAGGCTTAGTTGAGAATGAAGAC |
| *CBL* | Sugarcane_Unigene_BMK.25350 | TGCCTGGATGGAGTGAAG | GCTCATACAACGCCTCAAC |
| *MDAR* | Sugarcane_Unigene_BMK.69164 | TCGCCATCATCTCCAAGG | GTACTCAGGATCAGTTCAATGC |
| *Prx* | Sugarcane_Unigene_BMK.42965 | CCTCCACCTCTGCGTTCC | CTTGCCGTTCTGGTCCTTGA |
| *RPM1* | Sugarcane_Unigene_BMK.66398 | GGATGACTGAGGAGAAGAAGAT | CACTCTGGTGGAGAATTGTTG |
| *PBS1* | Sugarcane_Unigene_BMK.67719 | TGACTGCTCTGAGGTGTATC | CTCCTGCCACTGTTATTCTTG |
| *HSP90* | Sugarcane_Unigene_BMK.42924 | TTGTTAAGGGTATTGTTGACTCTG | ACACTTCTTCACGAGGTTCT |
| *HSP90* | gi35034166 | CCTCATCATCAACACCTTCTACTC | ACTTGTCCGTCAGGCTCTC |
| *PP2C* | Sugarcane_Unigene_BMK.63236 | ATGTGGTCTCTAACGAGGAAG | GTTATGTTGTCACTGCTCTCC |
| *ACO* | gi35014290 | CACCAAGTTCGCCATGTAC | CTGGAGGAGCAGGATGATG |
| *EIN3* | Sugarcane_Unigene_BMK.65773 | CCTCGTTCGTCAGTCCAA | GCTCCTCTTCCTGCTTGA |
| *GID1* | Sugarcane_Unigene_BMK.57806 | CCATCCTCGTCTACTACCAC | TGTTGAAGTAGGCGTGGAA |
| *GAPDH* | - | CACGGCCACTGGAAGCA | TCCTCAGGGTTCCTGATGCC |

Notes: *POD*, peroxidase; *PR1*, pathogenesis-related protein 1; *HSP*, heat shock protein; *CaM*, calmodulin; *CML*, calcium-binding protein; *CaMBP*, calmodulin-binding protein; *CBL*, calcineurin B-like protein; *MDAR*, monodehydroascorbic acid reductase; *Prx*, peroxiredoxin; *RPM1*, effector-triggered immune receptor; *PBS1*, serine-threonine kinase; *HSP90*, heat shock protein 90; *PP2C*, protein phosphatase 2C; *ACO*, 1-aminocycopropane-1-carbosylic acid oxidase; *EIN3*, ethylene sensitive 3; *GID1*, gibberellic acid-insensitive dwarf 1; *GAPDH*, glyceraldehyde-3-phosphate dehydrogenase.
