# Supplementary material for: Comparative proteomics reveals that central metabolism changes are associated with resistance against Sporisorium scitamineum in sugarcane
Source: BMC Genomics. 2016 Oct 12;17:800. doi: 10.1186/s12864-016-3146-8 (PMC5062822; doi:10.1186/s12864-016-3146-8)
Supplement: Additional file 3: Figure S1. — The detection of gDNA PCR and total RNA RT-PCR of resistance plants for ScGluA1 gene. M, DNA marker 15,000 + 2000 bp; 1 ~ 5, The gDNA PCR amplification products of transgenic plants; 1’ ~ 5’, The total RNA RT-PCR amplification products of transgenic plants; 6 and 6’, The amplification products of pCAMBIA 1301-ScGluA1; 7 and 7’, The amplification products of non-transgenic plants; 8 and 8’, Blank control. (DOCX 242 kb) [file 12864_2016_3146_MOESM3_ESM.docx]

**Comparative proteomics reveals that central metabolism changes are associated with resistance against *Sporisorium scitamineum* in sugarcane**

**Yachun** **Su^1^**

**E-mail:** **[syc2009mail@163.com](mailto:syc2009mail@163.com)**

**Liping Xu^1^***

**E-mail:** [**xlpmail@126.com**](mailto:xlpmail@126.com)

**Zhuqing Wang^1^**

**E-mail:** [**zhuqingemail@163.com**](mailto:zhuqingemail@163.com)

**Qiong Peng^1^**

**E-mail:** [**pengqiongfj@163.com**](mailto:pengqiongfj@163.com)

**Yuting Yang^1^**

**E-mail:** **[yytjiayou@126.com](mailto:yytjiayou@126.com)**

**Yun** **Chen^1^**

**E-mail:** **[sweetchenyun@163.com](mailto:sweetchenyun@163.com)**

**Youxiong Que^1,2^***

**E-mail:** [**queyouxiong@126.com**](mailto:queyouxiong@126.com)

^1^Key Laboratory of Sugarcane Biology and Genetic Breeding, Ministry of Agriculture, Fujian Agriculture and Forestry University, Fuzhou 350002, China

^2^Guangxi Collaborative Innovation Center of Sugarcane Industry, Guangxi University, Nanning 530005, China

***Correspondence should be addressed to** [xlpmail@126.com](mailto:xlpmail@126.com) and [queyouxiong@126.com](mailto:queyouxiong@126.com)

**The full postal address of the submitting author Youxiong Que is as follows:** Key Laboratory of Sugarcane Biology and Genetic Breeding, Ministry of Agriculture, Fujian Agriculture and Forestry University, Fuzhou 350002, China


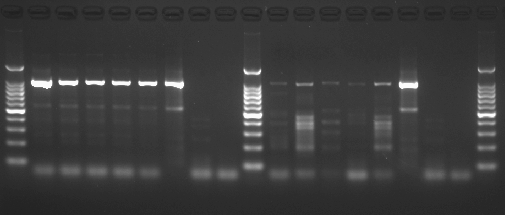


M 1 2 3 4 5 6 7 8 M 1’ 2’ 3’ 4’ 5’ 6’ 7’ 8’ M

1500 bp

1000 bp

1500 bp

1000 bp

Additional file 3: **Figure S1** The detection of gDNA PCR and total RNA RT-PCR of resistance plants for *ScGluA1* gene. M, DNA marker 15,000+2,000 bp; 1~5, The gDNA PCR amplification products of transgenic plants; 1’~5’, The total RNA RT-PCR amplification products of transgenic plants; 6 and 6’, The amplification products of pCAMBIA 1301-*ScGluA1*; 7 and 7’, The amplification products of untransgenic plants; 8 and 8’, Blank control.
