# Supplementary material for: Comparative proteomics reveals that central metabolism changes are associated with resistance against Sporisorium scitamineum in sugarcane
Source: BMC Genomics. 2016 Oct 12;17:800. doi: 10.1186/s12864-016-3146-8 (PMC5062822; doi:10.1186/s12864-016-3146-8)
Supplement: Additional file 9: Table S7. — Transitions of five differentially expressed proteins selected for the MRM validation. (DOCX 20 kb) [file 12864_2016_3146_MOESM9_ESM.docx]

**Comparative proteomics reveals that central metabolism changes are associated with resistance against *Sporisorium scitamineum* in sugarcane**

**Yachun** **Su^1^**

**E-mail:** **[syc2009mail@163.com](mailto:syc2009mail@163.com)**

**Liping Xu^1^***

**E-mail:** [**xlpmail@126.com**](mailto:xlpmail@126.com)

**Zhuqing Wang^1^**

**E-mail:** [**zhuqingemail@163.com**](mailto:zhuqingemail@163.com)

**Qiong Peng^1^**

**E-mail:** [**pengqiongfj@163.com**](mailto:pengqiongfj@163.com)

**Yuting Yang^1^**

**E-mail:** **[yytjiayou@126.com](mailto:yytjiayou@126.com)**

**Yun** **Chen^1^**

**E-mail:** **[sweetchenyun@163.com](mailto:sweetchenyun@163.com)**

**Youxiong Que^1,2^***

**E-mail:** [**queyouxiong@126.com**](mailto:queyouxiong@126.com)

^1^Key Laboratory of Sugarcane Biology and Genetic Breeding, Ministry of Agriculture, Fujian Agriculture and Forestry University, Fuzhou 350002, China

^2^Guangxi Collaborative Innovation Center of Sugarcane Industry, Guangxi University, Nanning 530005, China

***Correspondence should be addressed to** [xlpmail@126.com](mailto:xlpmail@126.com) and [queyouxiong@126.com](mailto:queyouxiong@126.com)

**The full postal address of the submitting author Youxiong Que is as follows:** Key Laboratory of Sugarcane Biology and Genetic Breeding, Ministry of Agriculture, Fujian Agriculture and Forestry University, Fuzhou 350002, China

**Additional file 9: Table S7** Transitions of five differentially expressed proteins selected for the MRM validation

| **NO** | **Protein Name** | **Protein ID** | **Peptide sequence** | **Precursor ion (m/z) (Q1)** | **Product ion (m/z) (Q3)** | **Dwell time**  **(ms)** | **CE (V)** |
| --- | --- | --- | --- | --- | --- | --- | --- |
| 1 | Prx | Sugarcane_Unigene_BMK.42965 | LPFTLLSDDGNR | 674.35 | 1234.61 | 10 | 33.1 |
|  |  |  |  | 674.35 | 1137.55 | 10 | 33.1 |
|  |  |  |  | 674.35 | 990.49 | 10 | 33.1 |
|  |  |  |  | 674.35 | 889.44 | 10 | 33.1 |
|  |  |  |  | 674.35 | 776.35 | 10 | 33.1 |
|  |  |  |  | 674.35 | 663.27 | 10 | 33.1 |
| 2 | CCaOMT | Sugarcane_Unigene_BMK.55608 | VIAFDVSR | 453.76 | 807.44 | 10 | 25.2 |
|  |  |  |  | 453.76 | 694.35 | 10 | 25.2 |
|  |  |  |  | 453.76 | 623.31 | 10 | 25.2 |
|  |  |  |  | 453.76 | 476.25 | 10 | 25.2 |
|  |  |  |  | 453.76 | 361.22 | 10 | 25.2 |
|  |  |  |  | 453.76 | 213.16 | 10 | 25.2 |
| 3 | 4CL | Sugarcane_Unigene_BMK.69390 | LLITNAEGNAAAQAAAGK | 842.45 | 1243.60 | 10 | 39.2 |
|  |  |  |  | 842.45 | 1129.56 | 10 | 39.2 |
|  |  |  |  | 842.45 | 1058.52 | 10 | 39.2 |
|  |  |  |  | 842.45 | 929.48 | 10 | 39.2 |
|  |  |  |  | 842.45 | 687.38 | 10 | 39.2 |
|  |  |  |  | 842.45 | 626.39 | 10 | 39.2 |
|  |  |  | FGWFHTGDIGVVDEEGYVR | 728.34 | 966.45 | 10 | 37.2 |
|  |  |  |  | 728.34 | 867.38 | 10 | 37.2 |
|  |  |  |  | 728.34 | 623.31 | 10 | 37.2 |
|  |  |  |  | 728.34 | 494.27 | 10 | 37.2 |
|  |  |  |  | 728.34 | 1118.51 | 10 | 37.2 |
|  |  |  |  | 728.34 | 1217.57 | 10 | 37.2 |
| 4 | CCR | Sugarcane_Unigene_BMK.47942 | VVHYSELVNIIR | 481.28 | 614.40 | 10 | 23.8 |
|  |  |  |  | 481.28 | 515.33 | 10 | 23.8 |
|  |  |  |  | 481.28 | 288.20 | 10 | 23.8 |
|  |  |  |  | 481.28 | 336.20 | 10 | 23.8 |
|  |  |  |  | 481.28 | 715.34 | 10 | 23.8 |
|  |  |  |  | 481.28 | 828.43 | 10 | 23.8 |
|  |  |  | SLGIELIPVETSVK | 742.93 | 1114.64 | 10 | 35.6 |
|  |  |  |  | 742.93 | 985.59 | 10 | 35.6 |
|  |  |  |  | 742.93 | 872.51 | 10 | 35.6 |
|  |  |  |  | 742.93 | 759.42 | 10 | 35.6 |
|  |  |  |  | 742.93 | 258.14 | 10 | 35.6 |
|  |  |  |  | 742.93 | 613.35 | 10 | 35.6 |
| 5 | POD | Sugarcane_Unigene_BMK.73465 | ASLEAQCPGVVSCADILAFAAR | 1153.57 | 1194.59 | 10 | 50.4 |
|  |  |  |  | 1153.57 | 761.47 | 10 | 50.4 |
|  |  |  |  | 1153.57 | 648.38 | 10 | 50.4 |
|  |  |  |  | 1153.57 | 600.30 | 10 | 50.4 |
|  |  |  |  | 1153.57 | 760.33 | 10 | 50.4 |
|  |  |  |  | 1153.57 | 1013.47 | 10 | 50.4 |
|  |  |  | ALVDSFVR | 453.76 | 722.38 | 10 | 25.2 |
|  |  |  |  | 453.76 | 623.31 | 10 | 25.2 |
|  |  |  |  | 453.76 | 508.29 | 10 | 25.2 |
|  |  |  |  | 453.76 | 421.26 | 10 | 25.2 |
|  |  |  |  | 453.76 | 284.20 | 10 | 25.2 |
| 6 | beta-galactosidase | P00722 | VDEDQPFPAVPK | 671.34 | 998.53 | 10 | 33.0 |
|  |  |  |  | 671.34 | 755.45 | 10 | 33.0 |
|  |  |  |  | 671.34 | 511.32 | 10 | 33.0 |
|  |  |  |  | 671.34 | 587.23 | 10 | 33.0 |
|  |  |  | APLDNDIGVSEATR | 729.37 | 1176.55 | 10 | 35.1 |
|  |  |  |  | 729.37 | 1061.52 | 10 | 35.1 |
|  |  |  |  | 729.37 | 832.45 | 10 | 35.1 |
|  |  |  |  | 729.37 | 719.37 | 10 | 35.1 |
|  |  |  |  | 729.37 | 693.85 | 10 | 35.1 |
|  |  |  | IDPNAWVER | 550.28 | 986.47 | 10 | 28.7 |
|  |  |  |  | 550.28 | 871.44 | 10 | 28.7 |
|  |  |  |  | 550.28 | 774.39 | 10 | 28.7 |
|  |  |  |  | 550.28 | 660.35 | 10 | 28.7 |
|  |  |  |  | 550.28 | 589.31 | 10 | 28.7 |
|  |  |  |  | 550.28 | 403.23 | 10 | 28.7 |
|  |  |  | VNWLGLGPQENYPDR | 879.43 | 1245.59 | 10 | 40.5 |
|  |  |  |  | 879.43 | 1188.56 | 10 | 40.5 |
|  |  |  |  | 879.43 | 1075.48 | 10 | 40.5 |
|  |  |  |  | 879.43 | 1018.46 | 10 | 40.5 |
|  |  |  |  | 879.43 | 387.20 | 10 | 40.5 |
|  |  |  |  | 879.43 | 400.20 | 10 | 40.5 |
|  |  |  | GDFQFNISR | 542.26 | 911.47 | 10 | 28.4 |
|  |  |  |  | 542.26 | 764.41 | 10 | 28.4 |
|  |  |  |  | 542.26 | 636.35 | 10 | 28.4 |
|  |  |  |  | 542.26 | 489.28 | 10 | 28.4 |
|  |  |  |  | 542.26 | 262.15 | 10 | 28.4 |
|  |  |  |  | 542.26 | 320.12 | 10 | 28.4 |

Notes: Prx, peroxiredoxin; CCaOMT, caffeoyl CoA O-methyltransferase; 4CL, 4-coumarate CoA ligase; CCR, cinnamoyl CoA reductase; POD, peroxidase. Beta-galactosidase peptides were used as internal standards for relative quantification of protein levels. Q1 mean the mass to charge (m/z) ratio of the parent ion. Q3 mean the mass to charge (m/z) ratio of the product ion. CE represented collision energy.
